# SALIS transcriptionally represses IGFBP3/Caspase-7-mediated apoptosis by associating with STAT5A to promote hepatocellular carcinoma

**DOI:** 10.1038/s41419-022-05094-z

**Published:** 2022-07-23

**Authors:** Xingyuan Liu, Yi Jin, Xuan Wan, Xiaoting Liang, Ke Wang, Jieyu Liu, Jiale Jiang, Bingyao Meng, Shuo Han, Liang Zhou, Shaoxi Cai, Fei Zou

**Affiliations:** 1 Department of Occupational Health and Occupational Medicine, Guangdong Provincial Key Laboratory of Tropical Disease Research, School of Public Health, Southern Medical University, Guangzhou, China; 2 Department of Toxicology, Guangdong Provincial Key Laboratory of Tropical Disease Research, School of Public Health, Southern Medical University, Guangzhou, China; 3 Chronic Airways Diseases Laboratory, Department of Respiratory and Critical Care Medicine, Nanfang Hospital, Southern Medical University, Guangzhou, China

**Keywords:** Liver cancer, Liver cancer

## Abstract

Hepatocellular carcinoma (HCC) is the most common subtype of liver cancer and the second most fatal cancer in the world despite the great therapeutic advances in the past two decades, which reminds us of the gap in fully understanding the oncogenic mechanism of HCC. To explore the key factors contributing to the progression of HCC, we identified a LncRNA, termed SALIS (Suppression of Apoptosis by LINC01186 Interacting with STAT5A), functions in promoting the proliferation, colony formation, migration and invasion while suppressing apoptosis in HCC cells. Mechanistic study indicated SALIS physically associates with transcription factor STAT5A and binds to the promoter regions of IGFBP3 and Caspase-7 to transcriptionally repress their expression and further inhibit apoptosis. Our findings identified SALIS as an oncogene to promote HCC by physically binding with STAT5A to inhibit the expression of pro-apoptotic IGFBP3 and Caspase-7, which suggests novel therapeutic targets for HCC treatments.

## Introduction

As the second leading cause of tumor-related death, hepatocellular carcinoma (HCC) is the most common subtype of liver cancers with 746,000 death annually [1, 2] and estimated to be up to one million death in 2030 [3]. Virus infection and alcohol abuse account for the major risk factors of HCC, however, nonalcoholic fatty liver disease together with metabolic syndrome and obesity become the main cause of liver cancer gradually [3]. The mild symptoms of HCC in early and middle stages delayed the timely treatment and led to an unfavorable outcome that most of the HCC patients were diagnosed at an advanced stage. Under such circumstance, traditional treatments including ablation, resection, and liver transplantation may not be effective enough [4]. Thus, in-depth understanding the mechanisms of the oncogenesis and progression of HCC would be helpful to develop curative treatments.

Long noncoding RNAs (LncRNAs) are a class of transcripts with length exceeding 200 nucleotides (nt) and harboring no protein-coding potential. Hundreds of LncRNAs have been identified to play diverse roles in various cellular processes by acting as signaling molecules, decoys, guides, sponges for modulating miRNA function, or scaffolds for epigenetic regulation of gene expression [5]. During the past decade, abundant evidence supported LncRNAs are involved in tumor carcinogenesis and progression [6]. For example, MALAT1 promotes the development of cutaneous squamous cell carcinoma by associating with c-MYC to transcriptionally modulate KTN1 expression and indirectly regulate EGFR protein expression [7]. For HCC, current reports have disclosed the iceberg tip of the diverse roles of HCC-related LncRNAs. For example, stemness contributes to the initiation and progression of HCC. DANCR (Anti-differentiation noncoding RNA) is higher-expressed in stem-like HCC cells and facilitates the oncogenesis and intrahepatic/extrahepatic tumor colonization by associating with and liberating CTNNB1 mRNA from the repressing effect of CTNNB1-targetive miRNAs [8]. PSTAR (p53-stabilizing and activating RNA) is functionally inhibits HCC cell proliferation and tumorigenicity. Mechanistically, PSTAR binds to hnRNP K and enhances its SUMOylation to stabilize the interaction between p53 and hnRNP K for ultimately activating p53-mediated cell cycle arrest [9]. Of course, there are still a long way to decipher the full scope of the functions and mechanisms of LncRNAs in HCC.

Signal Transducers and Activators of Transcription (STAT) family is a group of intracellular transcription factors that regulate gene expression to influence cell survival, cell cycle and immune responses associated with cancer progression [10]. Total seven STAT family numbers exist in human including STAT1, STAT2, STAT3, STAT4, STAT5A, STAT5B and STAT6, despite STAT4 and STAT6 harbor limited roles in tumors [11]. Chronic inflammatory injury induced by virus (HBV or HCV) infection or chemicals exposure-caused hepatitis is one of the major reasons leading to HCC, during which inflammatory factors including interferons and interleukins can activate Janus Kinase (JAK)-STATs pathway [11]. STAT1 and STAT2 have been shown to inhibit HCV replication after being activated by IFNs [12], while activation of STAT1 and STAT3 are found in non-alcoholic steatohepatitis (NASH) [13]. Many studies indicate that STAT3-mediated pathway contributes to HCC metastasis and proliferation [14–16]. STAT5 commonly refers to two proteins, STAT5A and STAT5B, which share 94% structural homology but are transcribed from different gene loci. STAT5B was reported to be activated in HCC and its expression was significantly correlated with the aggressive tumor behaviors [17]. However, little is known about whether STAT5A is involved in HCC.

Insulin like growth factor binding protein 3 (IGFBP3) is a component of insulin-like growth factor (IGF) complex and regulates IGFs’ functions. IGFBP3 also possesses IGF-independent roles to influence tumor development by modulating cell apoptosis [18]. Low levels of IGFBP3 are tightly associated with the development of common malignancies [19]. Tumor suppressor p53 could upregulate the mRNA expression of IGFBP3 and thus induces cell apoptosis in prostate cancer cells [20, 21]. In breast cancer, IGFBP3 regulates apoptosis through increasing the ratio of proapoptotic (Bax) relative to antiapoptotic (Bcl-2) proteins [22]. Also, the pro-apoptotic role of IGFBP3 is tightly related to the activation of caspase cascade [23].

In this study, to identify LncRNAs contributing to HCC tumorigenesis and progression, we performed bioinformatic analysis utilizing dataset from NCBI GEO and TCGA databases and finally focused on LINC01186, which we termed as SALIS (Suppression of Apoptosis by LINC01186 Interacting with STAT5A). SALIS is significantly higher-expressed in HCC relative to normal hepatic tissues and cells and acts as an oncogene to promote the proliferation, colony formation, migration, and invasiveness of HCC cells while inhibiting apoptosis. Mechanistically, transcriptomic sequencing identified IGFBP3 to be transcriptionally repressed by SALIS, which relies on the physical association with STAT5A and binding to the promoter regions of IGFBP3 and Caspase-7 for inhibiting their expression and the following apoptosis. In summary, we identified an oncogenic and anti-apoptotic SALIS-regulated pathway to regulate HCC progression, which may present a series of novel targets for HCC therapy.

### Results

#### SALIS is higher-expressed in HCC tumors and cells

**Fig. 1:**
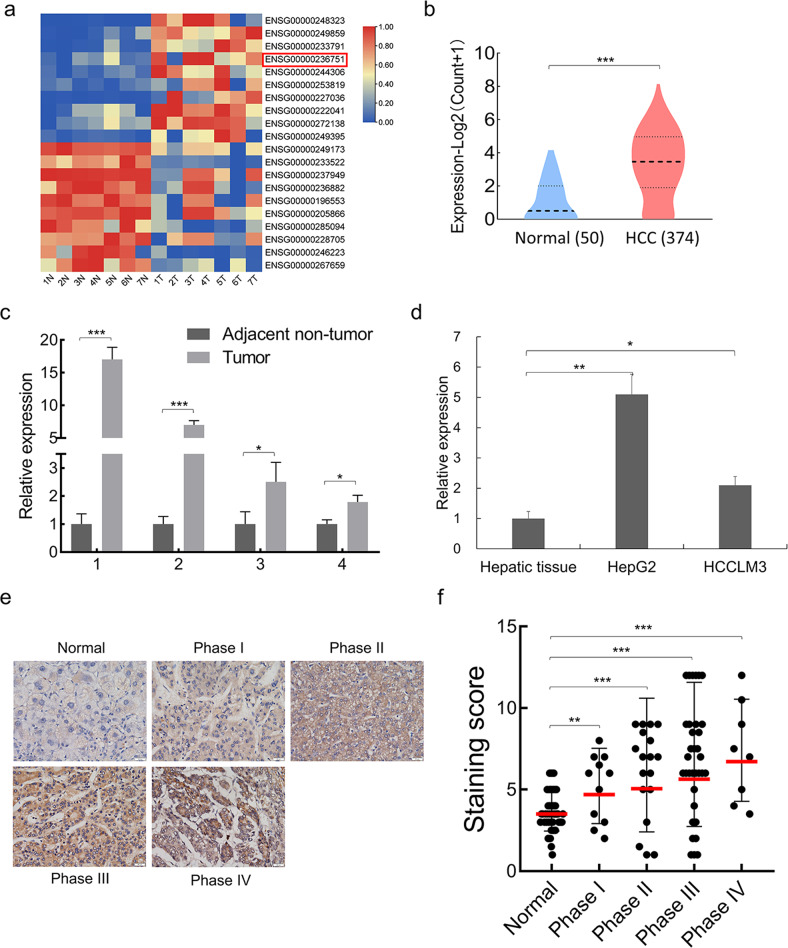
SALIS is higher-expressed in HCC tumors and cells. **a** The top 10 upregulated and downregulated LncRNAs from GSE101728 were clustered and shown in heat map. SALIS was highlighted using a red box. **b** The expression of SALIS in HCC (374 cases) compared with normal tissues (50 cases). The original expression data is from The Cancer Genome Atlas Program (TCGA) database. **c** Detection of the expression levels of SALIS in 4 paired clinicl samples of HCC and adjacent non-tumor tissues by qPCR. **d** Comparison of SALIS expression between normal hepatic tissue and HCC cell lines (HepG2 and HCCLM3) by qPCR. The qPCR data represent the average of three independent experiments ±s.d. **e** in situ hybridization (ISH) detection of SALIS in HCC tissue array and adjacent normal specimens. Representative images with various levels of staining (negative or weak from normal tissues, strong from tumor tissues) are shown. Scale bar: 50 μM. **f** Association of SALIS expression scores with AJCC tumor stages (Phase I, II, III, and IV). Data are plotted as the means of 95% confidence interval ± s.d. *P < 0.05, **P < 0.01, ***P < 0.001.

To explore LncRNAs that potentially influence HCC progression, we analyzed one published dataset (GSE101728) based on transcriptomic sequencing of HCC tumors (*n* = 7) and adjacent healthy tissues (*n* = 7) [24] and found 959 noncoding RNAs with significantly altered expression (L2FC > 2, *P*-value < 0.05), of which 257 were upregulated and 702 were downregulated in HCC. Next, we performed hierarchical clustering algorithm processing of the original normalized expression values of top ten highest-upregulated and downregulated LncRNAs respectively in HCC tumors (Fig. 1a). SALIS (LINC01186), one of the top five ranked LncRNAs, its significantly-higher expression in HCC was also validated using the expression data of 374 HCC tumor tissues and 50 normal tissues from The Cancer Genome Atlas program (TCGA) database (Fig. 1b) and thus was chosen for further analysis.

SALIS possesses two exons and produces a 655-nt transcript (Supplementary Fig. S1a). Practical verification of SALIS expression confirmed its relative higher expression in HCC tumors and cell lines (HepG2 and HCCLM3) (Fig. 1c, d). Further examination and scoring of SALIS expression in tissue microarray indicated a positive correlation with ascending HCC staging (American Joint Committee on Cancer, AJCC) (Fig. 1e), e.g., an increasing trend was observed across from normal tissue and the early AJCC staging of HCC (grade I & II) to late AJCC staging of HCC (grade III & IV) (Fig. 1f). Collectively, the above results suggested that SALIS is significantly upregulated in HCC cells and tissues and positively correlated with HCC staging.

#### SALIS promotes the proliferation, colony formation, migration, and invasiveness of HCC cells

**Fig. 2:**
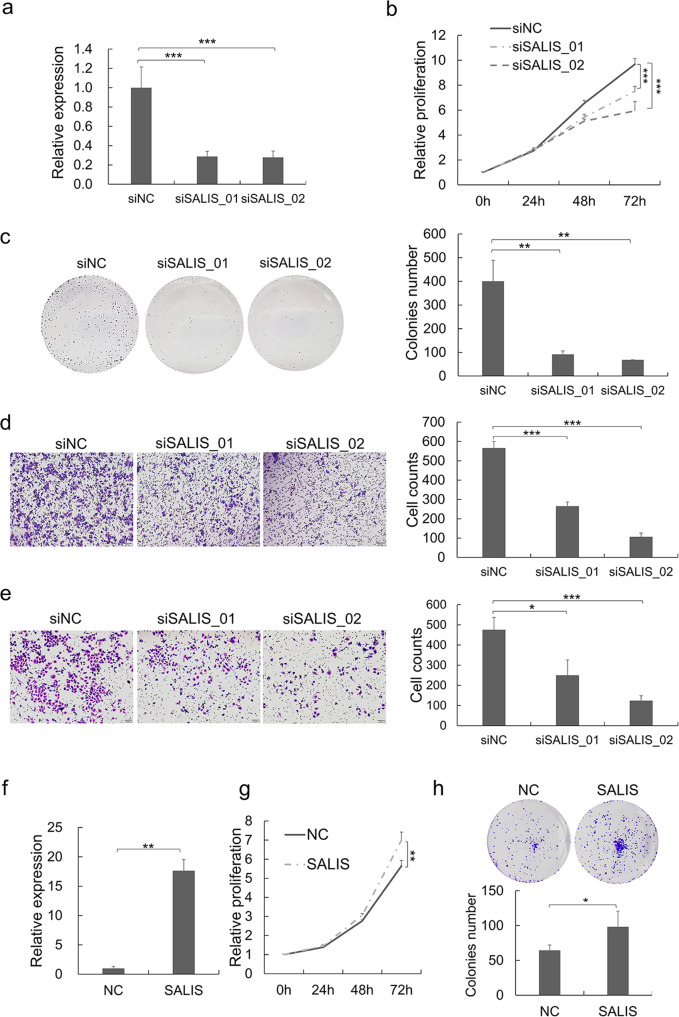
SALIS promotes cell proliferation, migration, and invasiveness in HCC cells. **a** SALIS RNA expression was detected by qPCR after knockdown of SALIS by siRNAs in HepG2 cells. Measurement of cell proliferation by CCK-8 assay (**b**), colony formation assay (**c**), transwell migration assay (**d**), and matrigel invasiveness measurement (**e**) were performed in HepG2 cells treated with siSALIS treatment. **f** SALIS RNA expression was detected by qPCR after overexpression of SALIS in HepG2 cells. Measurements of cell proliferation by CCK-8 assay (**g**), colony formation assay (**h**) were performed in HepG2 cells overexpressing SALIS. Data are plotted as the means ± s.d. *n* = 3. Scale bar: 20 μM. *P < 0.05, **P < 0.01, ***P < 0.001.

The expression levels of SALIS in different tissues and cancers are quite distinct (Supplementary Fig. S1b, c), indicating it may perform differential functions in cancers respectively. The higher expression level of SALIS in HCC tumors prompted us that SALIS probably functions as oncogene. To test this hypothesis, knockdown of SALIS was achieved by RNA interference (Fig. 2a and Supplementary Fig. S2a), which led to significant decreases of proliferative capacity (Fig. 2b and Supplementary Fig. S2b) and colony formation (Fig. 2c and Supplementary Fig. S2c) in HepG2 and HCCLM3 cells respectively. Further, Transwell migration assay and Matrigel invasiveness assay showed that SALIS depletion resulted in less cells penetrating the pores of the membrane (Fig. 2d) and weak capacity of invasiveness (Fig. 2e) compared with control group. Conversely, overexpression of SALIS in HepG2 cells promoted cell proliferation and colony formation (Fig. 2f–h).

Together, the above data indicated that SALIS functions as an oncogene to enhance tumor growth, colon formation, migration, and invasiveness in HCC cells.

#### Transcriptomic analysis reveals SALIS negatively regulates IGFBP3 and Caspase-7 expression

**Fig. 3:**
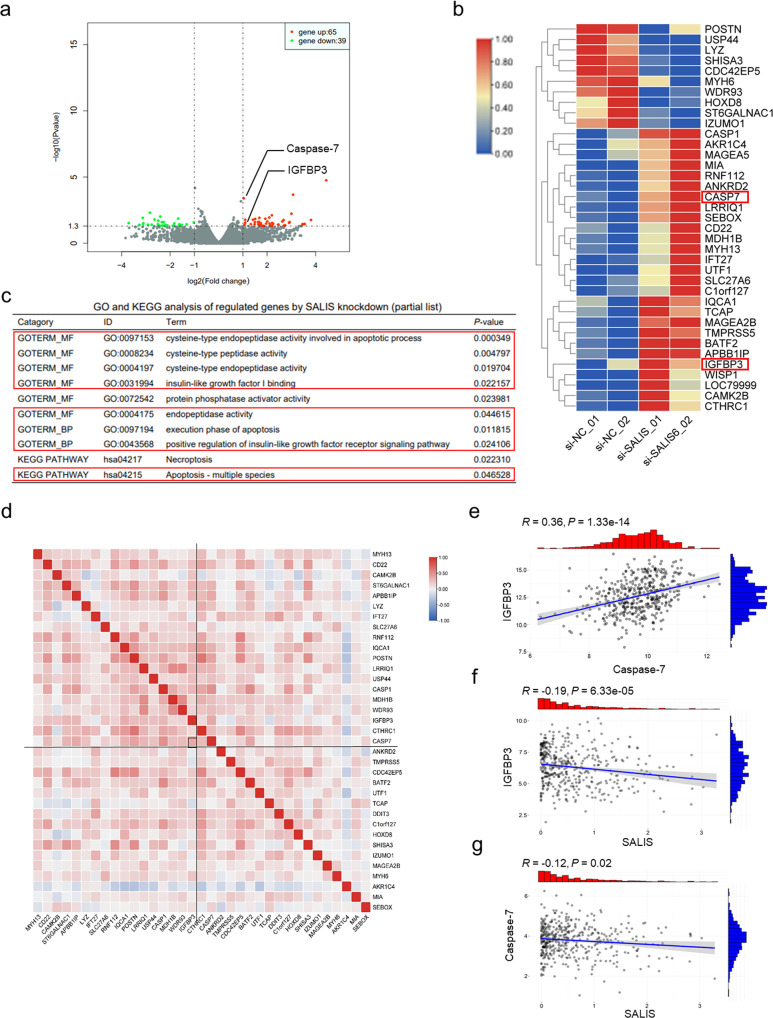
Genome-wide analysis of SALIS-regulated transcriptomic variations by RNA-Seq in HepG2 cells. **a** Total differentially-expressed genes (65 upregulated and 39 downregulated) between siNC-treated and siSALIS-treated HepG2 cell groups were determined by volcano plot. RNA-seq data of two biological repeats treated with same siNC but different siSALIS oligos were analyzed here. **b** Differentially-expressed protein-coding genes (27 upregulated and 10 downregulated by exlcuding noncoding RNAs) were clustered and shown in heat map. Color bars at the left represent gene clusters established through k-means clustering. **c** Gene Ontology (GO) and Kyoto Encyclopedia of Genes and Genomes (KEGG) analysis of the differentially-expressed genes after SALIS knockdown. Apoptosis and insulin-like growth factor-related categories are highlighted. **d** Correlation analysis of the differentially-expressed genes from our RNA-seq were performed using clinical data from TCGA. IGFBP3 and Caspase-7 are underlined. The significant correlationship of Caspase-7 and IGFBP3 are shown in (**e**) and the significant correlationship of SALIS and IGFBP3 or Caspase-7, are shown in (**f**) and (**g**) respectively.

To address the underlying mechanism of the oncogenic function of SALIS, RNA-seq analysis after SALIS knockdown in HepG2 cells was performed. According to the criteria (L2FC > 1, *P*-value < 0.05), 65 upregulated and 39 downregulated genes were identified in response to SALIS knockdown (Fig. 3a, b and Supplementary Table S1). By Kyoto Encyclopedia of Genes and Genomes (KEGG) and Gene Ontology (GO) functional and signaling pathway analysis of the above differentially-expressed genes, the results showed the top-ranked lists of enriched KEGG and GO categories including “cysteine-type endopeptidase activity involved in apoptotic process”, “cysteine-type peptidase activity”, “cysteine-type endopeptidase activity”, “insulin-like growth factor I binding”, “protein phosphatase activator activity”, “endopeptidase activity”, “execution phase of apoptosis”, “positive regulation of insulin-like growth factor receptor signaling pathway”, “Necroptosis”, “Apoptosis - multiple species” (Fig. 3c and Supplementary Table S2). From the above categories, the role of SALIS appears tightly related with apoptosis.

Correlation analysis of the significantly-regulated genes from the above categories in TCGA database identified two upregulated and apoptosis-related genes in response to SALIS depletion, IGFBP3 and Caspase-7, which expressions are positively correlated (*R* = 0.36, *P* = 1.33e−14) (Fig. 3d, e). At the same time, significantly negative correlations between SALIS and IGFBP3 (*R* = −0.19, *P* = 6.33e−05) or Caspase-7 (*R* = −0.12, *P* = 0.02) were also obtained respectively (Fig. 3f, g).

Collectively, the above results suggested that SALIS might negatively regulate IGFBP3 and Caspase-7 in HCC.

#### SALIS represses apoptosis through IGFBP3 and Caspase-7

**Fig. 4:**
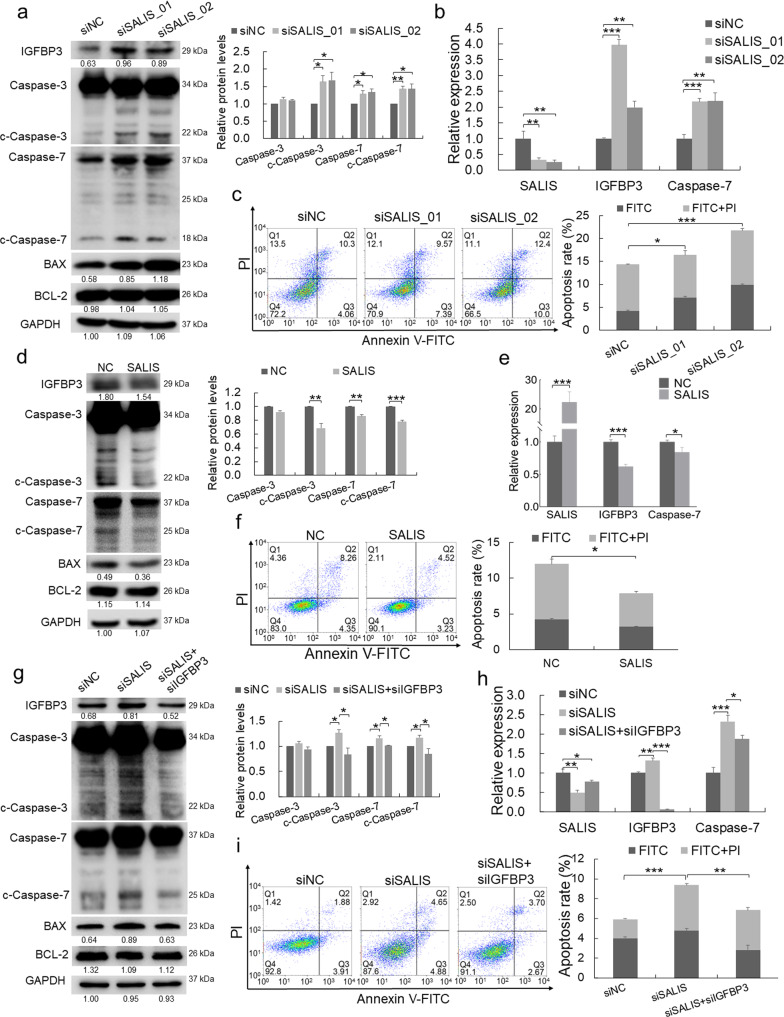
SALIS represses apoptosis through IGFBP3 and Caspase-7. **a** Protein levels of IGFBP3, BAX, BCL-2, Caspase-7, and cleavages of Caspase-3 and Caspase-7 were detected in HepG2 cells by Western blot after SALIS knockdown. GAPDH was using as loading control. The bar figure in right panel shows the statistically-analyzed relative protein levels of Caspase-3, Caspase-7 and their cleaved forms. **b** SALIS, IGFBP3, and Caspase-7 RNA expression were detected by qPCR after knockdown of SALIS by siRNAs in HepG2 cells. **c** Apoptosis was detected by flow cytometry with Annexin V/PI double staining in HepG2 cells after depletion of SALIS. The bar figure in right panel shows the statistically-analyzed apoptosis rate. **d** Protein levels of IGFBP3, BAX, BCL-2, Caspase-7, and cleavages of Caspase-3 and Caspase-7 were detected in HepG2 cells by Western blot after overexpressing SALIS. The bar figure in right panel shows the statistically-analyzed relative protein levels of Caspase-3, Caspase-7 and their cleaved forms. **e** SALIS, IGFBP3, and Caspase-7 RNA expression were measured by qPCR after SALIS overexpression in HepG2 cells. **f** The flow cytomety results in Annexin V/PI double stained HepG2 cells of NC or overexpressing SALIS. The bar figure in right panel shows the statistically-analyzed apoptosis rate. **g** Western blot of IGFBP3, BAX, BCL-2, Caspase-7, and cleavages of Caspase-3 and Caspase-7 in HepG2 cells treated with siNC, siSALIS or both of siSALIS and siIGFBP3. The bar figure in right panel shows the statistically-analyzed relative protein levels of Caspase-3, Caspase-7 and their cleaved forms. **h** The qPCR results of SALIS depletion and double knockdown of IGFBP3 and SALIS in HepG2 cells. **i** The distributions of HepG2 cells stained with Annexin V/PI in siNC, siSALIS, or siSALIS+siIGFBP3 treatment. The bar figure in right panel shows the statistically-analyzed apoptosis rate. Data are plotted as the means ± s.d. *n* = 3. *P < 0.05, **P < 0.01, ***P < 0.001.

From the above analysis, we guess SALIS may potentially regulate apoptosis through IGFBP3 and Caspase-7. To examine this hypothesis, we verified their expression relationship and performed apoptosis-related analysis. Knockdown of SALIS induced the expression of IGFBP3 and Caspase-7 in both RNA and protein levels (Fig. 4a, b and Supplementary Fig. S3a, b) and enhanced the expression of BAX and the cleavage of both Caspase-3 and Caspase-7 compared with siNC group while BCL-2 expression was not influenced (Fig. 4a and Supplementary Fig. S3a). Concurrently, fluorescence-activated cell sorting (FACS) analysis with Annexin V-FITC/PI double staining showed that SALIS depletion promoted both of the early and late apoptosis in HCC cells (Fig. 4c and Supplementary Fig. S3c). Conversely, overexpression of SALIS downregulated the protein and RNA expression of IGFBP3 and Caspase-7, reduced the expression of BAX and inhibited the cleavage of Caspase-3 and Caspase-7 (Fig. 4d, e) and less of early and late apoptosis (Fig. 4f), while BCL-2 expression kept stable (Fig. 4d).

Since IGFBP3 is a pro-apoptotic factor, we further knocked down of IGFBP3 in addition to SALIS depletion (Fig. 4h), which treatment reduced the BAX expression and cleavages of Caspase-3 and Caspase-7 (Fig. 4g and Supplementary Fig. S3d) and suppressed SALIS depletion-induced apoptosis (Fig. 4i and Supplementary Fig. S3e). Meanwhile, double knockdown of SALIS and Caspase-7 (Supplementary Fig. S3g) was conducted and detected by Western blot and FACS assays. The protein level of Caspase-7, cleavages of Caspase3/7, and apoptosis rate in double knockdown group were decreased compared with single SALIS-depletion group (Supplementary Fig. S3f–h).

Taken together, this part of results reveals that SALIS functions in inhibiting apoptosis through negative regulation of IGFBP3 and Caspase-7 in HCC cells.

#### SALIS directly associates with STAT5A and binds to the promoter regions of IGFBP3 and Caspase-7

**Fig. 5:**
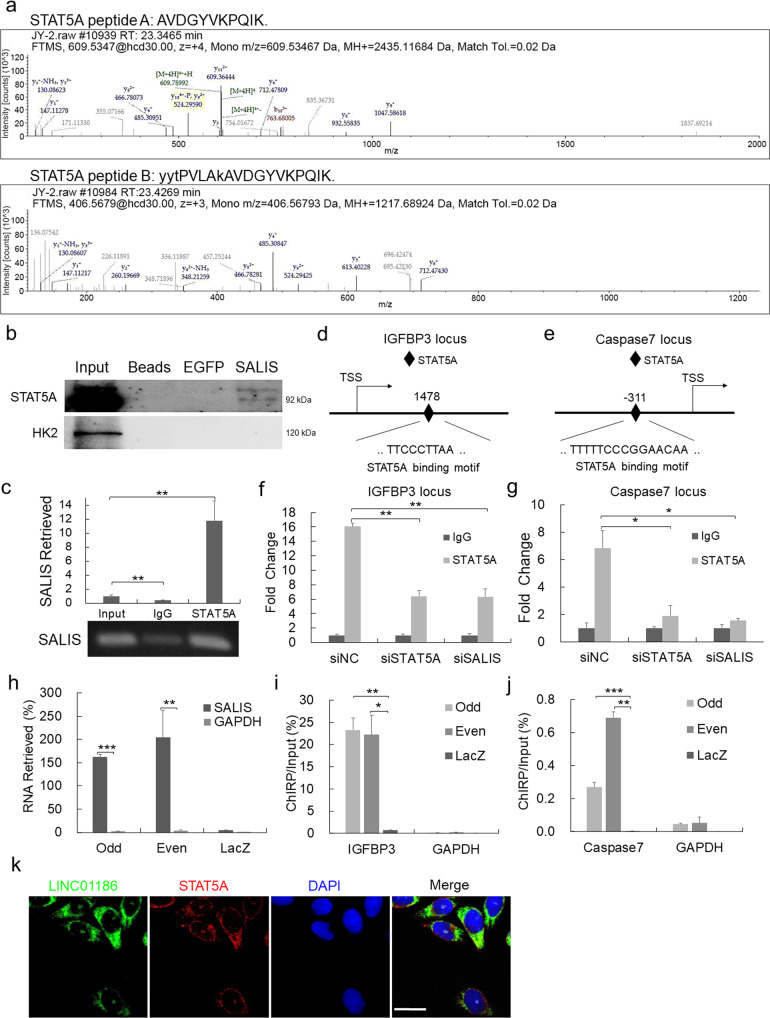
SALIS associates with STAT5A to transcriptionally regulate the expression of IGFBP3 and Caspase-7. **a** Two peptides were identified by high performance liquid chromatography-mass spectrometry (HPLC-MS) analysis of the protein mix precipitated by in vitro-transcribed Biotin-labeled SALIS RNA. **b** SALIS RNA transcript was used to retrieve STAT5A protein by RNA pull-down assay with beads only and EGFP as controls. The resulting protein mix was applied to detect STAT5A and HK2 by western blot. **c** RNA immunoprecipitation (RIP) assay was conducted using antibodies against STAT5A and IgG as control. **d**, **e** Predicted binding site of STAT5A (diamond) at the promoter regions of IGFBP3 and Caspase-7 by UCSC genome browser (http://genome.ucsc.edu/) and rVista (https://rvista.dcode.org/). **f**, **g** The binding enrichment of STAT5A at the above binding sites on the promoter regions of IGFBP3 and Caspase-7 was detected by ChIP-PCR after knockdown of SALIS or STAT5A. **h** Chromatin isolation by RNA purification (ChIRP) assay with both even and odd antisense oligos tiling SALIS shows the degree and specificity of SALIS retrieval. LacZ tiling oligo retrieval of SALIS RNA was used as a negative control. **i**, **j** Relative retrieval of genomic DNAs corresponding to the IGFBP3 or Caspase-7 promoter or GAPDH loci following SALIS or LacZ ChIRP. **k** Co-localization of SALIS with STAT5A in HepG2 cells was detected using FISH for SALIS and IF staining for STAT5A and observed by confocal microscope. Scale bar: 20 μM. The data are presented as means ± s.d., *n* = 3. *P < 0.05, **P < 0.01, ***P < 0.001.

LncRNAs commonly regulate gene transcription by associating with transcriptional factors and recruiting them to specific gene loci in genome to activate or inhibit gene expression [25, 26]. Given the mRNA expression of IGFBP3 and Caspase-7 is negatively responded to SALIS expression, we assumed a potential transcriptional regulatory mechanism may exist. Thus, we conducted an RNA pulldown assay using in vitro-transcribed full-length SALIS RNA accompanied with a control EGFP RNA. The specific binding proteins of SALIS was identified using high-performance liquid chromatography-mass spectrometry (HPLC-MS). Among the identified proteins, we focused on a highly-expressed transcription factor in HCC (Supplementary Fig. S4b), STAT5A, with two specific peptides charactered (Fig. 5a, Supplementary Fig. S4a, and Supplementary Table S3). The specific binding between SALIS and STAT5A was also verified by Western blot detection of the precipitated protein mix with a detection of irrelevant HK2 as control and RNA immunoprecipitation (RIP) (Fig. 5b, c). Further, the specific binding sites of STAT5A on the promoter regions of IGFBP3 (Fig. 5d) and Caspase-7 (Fig. 5e) were predicted by the UCSC genome browser (http://genome.ucsc.edu/) and rVista (https://rvista.dcode.org/). Subsequent validations using chromatin immunoprecipitation (ChIP) demonstrated that the binding enrichment of STAT5A at the promoters of IGFBP3 and Caspase-7 was significantly decreased in response to STAT5A knockdown (Fig. 5f, g). In addition, depletion of SALIS significantly reduced the binding enrichment of STAT5A at IGFBP3 and Caspase-7 promoters while did not influence STAT5A protein expression (Fig. 5f, g and Supplementary Fig. S4c). The above data suggests the specific enrichment of STAT5A at IGFBP3 and Caspase-7 promoter regions is dependent on SALIS.

To further analyze the interplays between SALIS and its target genes, we predicted the binding motif of SALIS on IGFBP3 and Caspase-7 promoter regions using LongTarget (http://lncrna.smu.edu.cn/) [27]. Next, we performed chromatin isolation by RNA purification (ChIRP) assay with both Odd and Even tiling oligos against SALIS and found that endogenous SALIS RNA but not GAPDH RNA was retrieved from chromatin, whereas negative control LacZ tiling oligos retrieved almost no SALIS RNA (Fig. 5h). Simultaneously, the Odd and Even oligos of SALIS significantly retrieved stronger amount of genomic DNA containing IGFBP3 and Caspase-7 loci than LacZ group while both Odd and Even group retrieve no signal of GAPDH locus (Fig. 5i, j). In short, SALIS physically and specifically binds to the promoter regions of IGFBP3 and Caspase-7. Further, RNA fluorescence in situ hybridization (FISH) and Immunofluorescence (IF) was performed. Though the subcellular location of SALIS and STAT5A are mainly in cytoplasm, we also observed significant portion of SALIS signal is co-localized with STAT5A in nucleus (Fig. 5k), which supports the transcriptional regulatory role of SALIS and STAT5A in nucleus.

Together, the above results suggest SALIS physically associates with STAT5A and binds to the promoter regions of IGFBP3 and Caspase-7.

#### The repression of IGFBP3 and Caspase-7 by SALIS is dependent on STAT5A

**Fig. 6:**
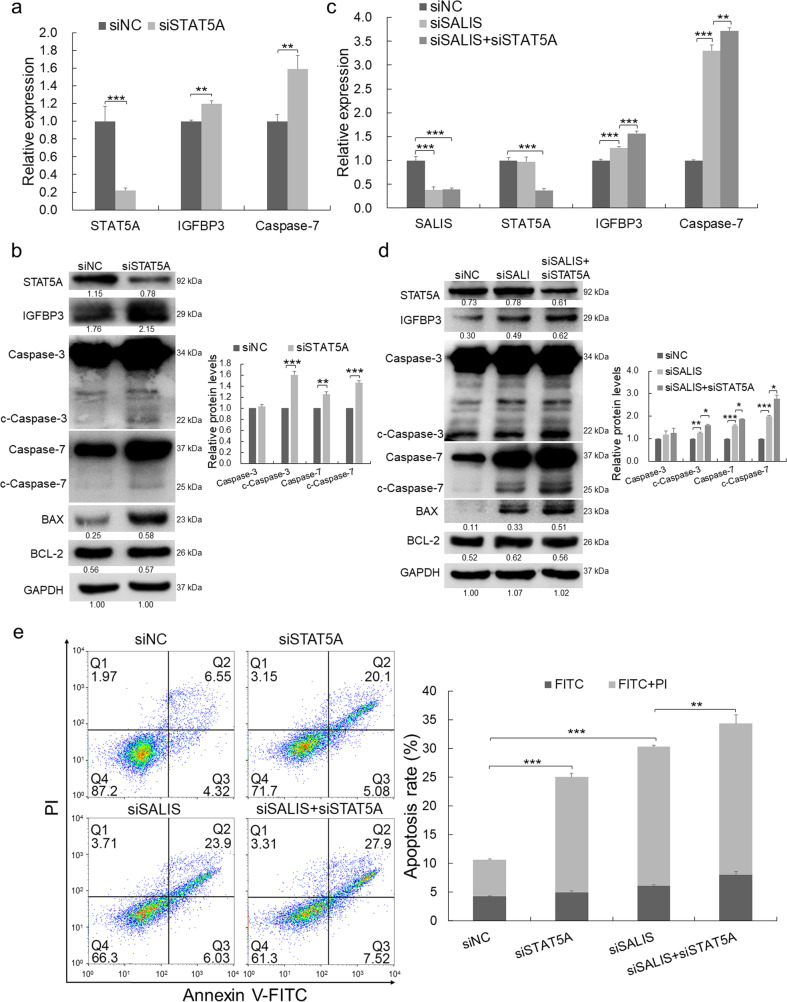
The repression of IGFBP3 and Caspase-7 and apoptosis by SALIS is dependent on STAT5A. **a** The RNA expression levels of STAT5A, IGFBP3, and Caspase-7 in HepG2 cells treated with siNC and siSTAT5A. **b** The protein levels of STAT5A, IGFBP3, BAX, BCL-2, Caspase-7, and cleaved forms of Caspase-3 and Caspase-7 were detected by Western blot after STAT5A knockdown. GAPDH was using as loading control. The bar figure in right panel shows the statistically-analyzed relative protein levels of Caspase-3, Caspase-7, and their cleaved forms. **c** The RNA expression levels of SALIS, STAT5A, IGFBP3, and Caspase-7 in HepG2 cells after treatment with siNC, siSALIS, siSALIS+siSTAT5A. **d** The protein levels of STAT5A, IGFBP3, BAX, BCL-2, Caspase-7, and cleaved forms of Caspase-3 and Caspase-7 were detected in HepG2 cells treated with siNC, siSALIS, siSALIS+siSTAT5A. The bar figure in right panel shows the statistically-analyzed relative protein levels of Caspase-3, Caspase-7 and their cleaved forms. **e** Apoptosis was detected by flow cytometry with Annexin V/PI double staining in HepG2 cells with treatment of siNC, siSTAT5A, siSALIS, siSALIS+siSTAT5A. The bar figure in right panel shows the statistically-analyzed apoptosis rate. The data are expressed as means ± s.d., *n* = 3. *P < 0.05, **P < 0.01, ***P < 0.001.

Our above results demonstrated SALIS may function to recruit STAT5A transcriptionally to regulate IGFBP3 and Caspase-7, in which STAT5A is the key mediator for fulfilling SALIS functions. To determine whether STAT5A is necessary for the inhibition of IGFBP3 and Caspase-7 expression by SALIS, we knocked down STAT5A and detected the mRNA and protein expression of IGFBP3 and Caspase-7. Loss of STAT5A resulted in significantly upregulation of IGFBP3 and Caspase-7 in both mRNA and protein levels together with enhanced generation of cleaved Caspase-3 and Caspase-7 and increased expression of BAX while BCL-2 expression was not influenced (Fig. 6a, b). Such results indicated STAT5A acts as a transcriptional factor for inhibiting the expression of IGFBP3 and Caspase-7. In addition, double-knockdown of both SALIS and STAT5A further induced the upregulation of IGFBP3 and Caspase-7 mRNA and protein expression, the expression of BAX, and the production of cleaved Caspase-3 and Caspase-7 compared to the single-knockdown of SALIS while BCL-2 expression kept stable (Fig. 6c, d). FACS assays also demonstrated double-depletion of both SALIS and STAT5A could induce more apoptosis compared with single-depletion of either SALIS or STAT5A (Fig. 6e).

Together, the above results suggest that STAT5A plays key role for SALIS to establish the transcriptional repression of IGFBP3 and Caspase-7 and inhibits apoptosis in HCC cells.

#### Loss of SALIS enhances apoptosis and compromises the growth of HCC in vivo

**Fig. 7:**
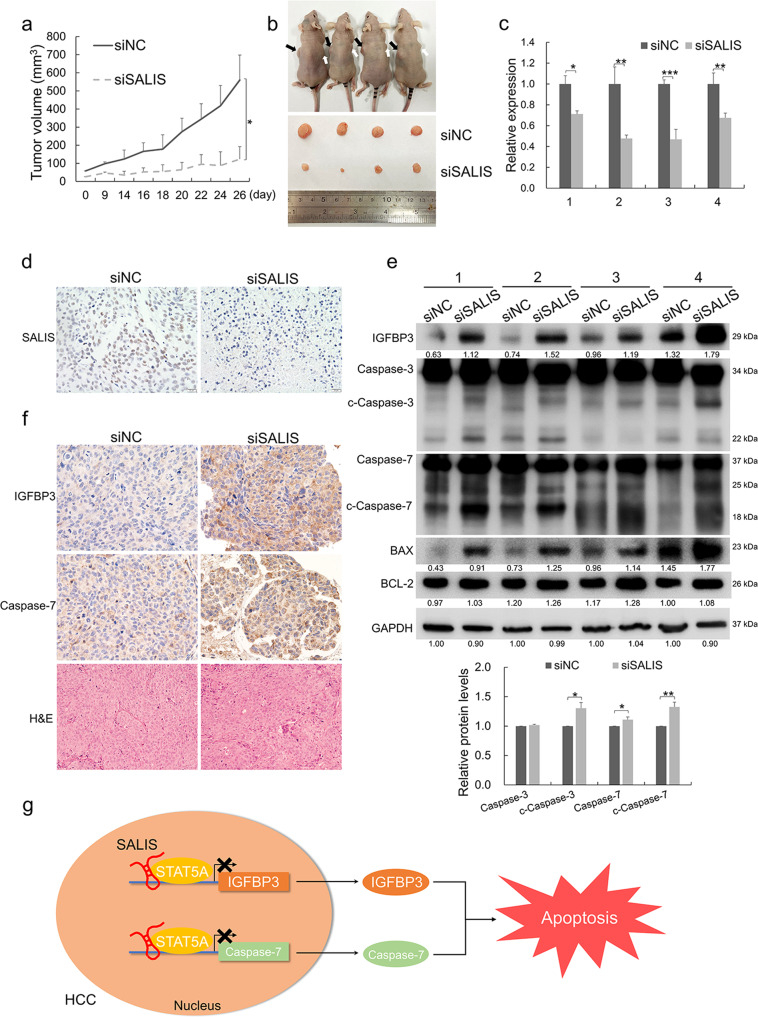
Loss of SALIS enhances apoptosis and compromises the growth of HCC in vivo. **a** Knockdown of SALIS inhibits tumor growth in mice xenograft model. Tumor volumes (mm^3^) were plotted according to day. **b** The mice were sacrificed at the end of the experiment. The upper panel shows the mice harboring xenograft tumors. Black arrows indicate the siNC-treated xenografts whereas white arrows indicate siSALIS-treated xenografts. The lower panel shows the dissected tumors from the experimental mice. **c** SALIS expression was detected in the dissected xenograft tumors by qPCR. **d** The expression levels of SALIS in tumor sections were evaluated using in situ hybridization to verify the efficiency of SALIS knockdown. **e** The protein levels of IGFBP3, BAX, BCL-2, cleavages of Caspase-3 and Caspase-7 were detected in SALIS-depleted groups together with paired control groups by Western blot. GAPDH was used as loading control. The bar figure in lower panel shows the statistically-analyzed relative protein levels of Caspase-3, Caspase-7, and their cleaved forms. **f** Immunohistochemical (IHC) staining of IGFBP3 and Caspase-7 expression of the xenograft tumors. The data are expressed as means ± s.d., *n* = 4. Scale bar: 20 μM. *P < 0.05, **P < 0.01, ***P < 0.001. **g** A model depicts SALIS interacts with STAT5A to transcriptionally modulate IGFBP3 and Caspase-7 expression to repress apoptosis and promote melanoma development.

To evaluate the pro-tumorigenic and anti-apoptotic functions of SALIS in vivo, we established a xenograft tumor model using immunocompromised nude mice. HCC cells treated with siNC or siSALIS were injected subcutaneously. There is less observable difference between the two groups initially. Gradually, SALIS knockdown significantly repressed the tumor growth compared with the control group and finally led to an apparent smaller xenograft mass at the end of the evaluation period (Fig. 7a, b). The loss of SALIS by in vivo RNA interference is validated by qPCR detections and ISH staining (Fig. 7c, d). Furthermore, knockdown of SALIS led to a significant upregulation of IGFBP3 and Caspase-7 expression (Fig. 7e, f) detected by Western blot and IHC (Immunohistochemistry) staining and generation of cleaved Caspase-3 and Caspase-7 together with the upregulation of BAX and relative stable expression of BCL-2 (Fig. 7e). Conversely, overexpression of SALIS significantly promoted tumor growth and formed bigger xenograft tumors (Supplementary Fig. S5a–c) and decreased IGFBP3 and Caspase-7 expression (Supplementary Fig. S5d, e) together with the reduction of BAX and generation of cleaved Caspase-3 and Caspase-7 (Supplementary Fig. S5d).

Taken together, the in vivo experiments demonstrated SALIS inhibits apoptosis by repressing IGFBP3 and Caspase-7 expression to promote HCC growth.

### Discussion

As one fundamental form of programmed cell death [28], apoptosis serves as a strong weapon of immune system arsenal to kill the virus-infected cells or transformed cells [29] and apoptotic cells commit suicide genetically by rapid demolition of cellular structures and organelles [30]. However, the modulation of apoptosis is defective in most types of cancer, in which dysregulation of apoptosis leads cancer cells to lose the ability of suicide [31]. In the past decades, tens of factors have been shown to be important player in apoptosis including cytokines, chemokines, transcriptional factors, and miRNAs [32, 33]. Yet, there are still large gap before fully understanding the regulatory network of apoptosis.

LncRNAs are novel players functioning in diverse pathophysiological processes and involved in the regulation of apoptosis. For example, LncRNA HOTAIR inhibits apoptosis in pancreatic cancer through interacting with EZH2 to upregulate the histone H3 lysine 27 trimethylation on death receptor 5 (DR5) gene and further inhibit DR5 expression [34]. In HCC, LINC01134 recruits SP1 to P62 gene promoter region and enhances its transcription which further activates the anti-oxidative stress response pathway and reduces apoptosis [35]. LncRNA HCP5 was identified to act as a scaffold for YB1 and ILF2, direct YB1 phosphorylation and translocation to nucleus, in which YB1 transcriptionally increases MSH5 expression by binding to promoter region of MSH5 and subsequently decreasing apoptosis by maintaining DNA double-strand breaks (DSBs) repair normally in premature ovarian insufficiency (POI) patients [36].

SALIS has previously been shown to be significantly lower-expressed in papillary thyroid carcinoma, nasopharyngeal carcinoma, and lung cancer and acts as tumor suppressor to inhibit tumor cell proliferation and migration [37–39], while potentially functions to repress apoptosis in nasopharyngeal carcinoma [38]. However, all the above reports of SALIS only carried out functional study and lack mechanist details. In this study, we demonstrated SALIS is higher-expressed in HCC, which is also supported by clinical data from TCGA database and contradictory to the tumor-suppressive role reported by the previous studies. As our transcriptomic analysis after SALIS depletion indicated the potential involvement of SALIS with apoptosis, we sought to explore the mechanism about how SALIS modulates apoptosis. KEGG and GO clustering analysis uncovered several apoptosis-related categories containing two upregulated key factors, IGFBP3 and Caspase-7.

There are two main pathways to regulate apoptosis: Death receptor pathway and mitochondria pathway [31]. Death receptor pathway is initiated by the tumor necrosis factors (TNF) binding to the TNF receptor (TNFR) on plasma membrane [40] and Bcl-2 family members are main modulatory proteins and responsible for regulating apoptosis following mitochondria pathway [41]. Although the two pathways are distinct and possess different key regulators to modulate apoptosis progression, they all converge to final caspase (cysteinyl aspartate-directed protease) cascade including Caspase-7 [40]. When apoptosis is executed, Caspase-3 and Caspase-7 are sequentially cleaved and activated by upstream caspase proteins. Further, the activated Caspase-3 and Caspase-7 cleaves a range of substrates, leading to the disassembly of the dying cells [42].

IGFBP3 was identified to promote apoptosis in cancers through several ways. In colon carcinoma, TP53 could upregulate the expression level of IGFBP3 and further promote apoptosis [20]. Additionally, IGFBP3 could induce Nur77 to translocate from nucleus to mitochondria and result in an activation of apoptotic cascade in many types of cancers [43]. Meanwhile, IGFBP3 competitively bound to GRP78 and disassociated GRP78 from Caspase-7, leading to enhanced production of activated Caspase-7 and the following apoptosis in breast cancer [44]. In this study, we identified the positive correlation of expression between IGFBP3 and Caspase-7 in HCC. Further, we demonstrated SALIS could transcriptionally suppress both of IGFBP3 and Caspase-7 expression to inhibit apoptosis.

STAT5A has been previously designed as a strategic target in the antitumor treatment with IMB-HDC, in which IMB-HDC plays a proapoptotic role by reducing STAT5A phosphorylation and nuclear translocation [45]. The suppression of STAT5A signaling pathway also promoted apoptosis in osteosarcoma [46]. In chronic myeloid leukemia (CML), STAT5A upregulated the expression level of miR-202–5p, which in turn inhibited USP15 expression to reduce Caspase-6 level and thereby lowered the apoptotic rate in CML cells [47]. Seeing IGFBP3 and Caspase-7 were upregulated in response to loss of SALIS, we tried to explore how SALIS regulates the expression of IGFBP3 and Caspase-7? With the hypothesis that SALIS may associates with a transcription factor to carry on its transcription regulatory function, we identified transcription factor STAT5A to directly interact with SALIS by RNA pulldown and HPLC-MS assays. Further ChIRP and ChIP assays demonstrated SALIS-STAT5A complex directly bound to the promoter regions of IGFBP3 and Caspase-7 genes to suppress apoptosis in HCC. Thus, our results are consistent with previous studies showing the involvement of STAT5A in apoptosis in tumors.

Collectively, our study identified SALIS is higher-expressed and functions as an oncogene to promote the proliferation, colony formation, migration, and invasiveness while repressing apoptosis in HCC. Transcriptomic sequencing after SALIS knockdown followed by KEGG and GO analysis found SALIS mainly regulates apoptotic pathway. The mechanistic study demonstrated that SALIS physically associates with STAT5A and binds to the promoter regions of IGFBP3 and Caspase-7 to transcriptionally repress their expressions and further inhibit apoptosis (Fig. 7g). Our findings highlight the oncogenic and anti-apoptotic functions of SALIS and established a novel SALIS-mediated and anti-apoptotic regulatory pathway, which suggests a series of novel intervention targets for HCC therapy.

## Methods

### Patient samples

This study was approved by the Institutional Review Board of Shanghai Outdo Biotech Co. Ltd. (Shanghai, China), all patients provided written informed consent for the use of surgical samples. The tissue array (HlivH150CS05) that included 75 HCC specimens and corresponding adjacent normal specimens was purchased from Shanghai Outdo Biotech Co. Ltd. Fresh HCC tissue samples and corresponding adjacent noncancerous tissues were obtained from patients diagnosed with HCC. None of the patients had been preprocessed with radiotherapy or chemotherapy before undergoing a hepatectomy.

### Cell lines

HCC lines HepG2 (Type Culture Collection of the Chinese Academy of Sciences, Shanghai, China), HCCLM3 (Procell Life Science & Technology Co., Ltd.) were cultured in Dulbecco’s modified Eagle medium (DMEM, Life Technologies) supplemented with 10% fetal bovine serum (ExCell Bio, FSP500) or Complete Classic Medium with Serum and CultureBoost™ (4Z0-500) and maintained at 37 °C with 5% CO_2_ in a humidified atmosphere. The STR profiles authentication information of all the cell lines used in this study were listed in Supplementary Figs. S5–6. All the cell lines have been tested and shown to be no mycoplasma contamination.

### Animal studies

Female athymic nude mice (BALB/C-nu/nu, 4–5 weeks old) purchased from the animal center of Southern Medical University were used for in vivo xenograft studies. The mice were euthanized by cervical dislocation to prevent suffering. This study was approved by the Institutional Animal Care and Use Committee (IACUC) of Nanfang Hospital affiliated with the Southern Medical University (Approval code L2019178). They are in accordance with the guidelines of the Asian Federation of Laboratory Animal Science Associations (AFLAS) and the National Regulations for the Administration of Affairs Concerning Experimental Animals (8 January 2011). Mouse transportation, housing, and breeding were conducted according to the recommendations of “The use of non-human animals in research.”

### RNA-seq data analysis

Total RNA was isolated from HepG2 using the Trizol Reagent (Ambion, USA) according to the manufacturer’s protocol, The quantity and integrity of RNA yield was assessed by using the K5500 (BeijingKaiao, China) and the Agilent 2200 TapeStation (Agilent Technologies, USA) separately. Briefly, the mRNA was enriched by oligodT conjugated NEBNext® Poly(A) mRNA Magnetic Isolation Module (NEB, USA) according to instructions. And then fragmented to be approximately 200 bp. Subsequently, the RNA fragments were subjected to first strand and second strand cDNA synthesis following by adaptor ligation and enrichment with a low-cycle according to instructions of NEBNext® Ultra™ RNA Library Prep Kit for Illumina. The purified library products were evaluated using the Agilent 2200 TapeStation and Qubit (Thermo Fisher Scientific, USA). The libraries were sequenced using Illumina (Illumina, USA) with paired-end 150 bp at Ribobio Co. Ltd (Ribobio, China).

### RNA isolation and quantitative real-time PCR (qRT-PCR)

Total RNAs from cells were extracted using TransZol reagent (TransGen Biotech Co., Ltd.) according to the manufacturer’s instructions. cDNAs were prepared using EasyScript All-in-One First-Strand cDNA Synthesis SuperMix for qPCR (One-Step gDNA Removal) (TransGen Biotech, AU341). mRNA expression analysis was performed using PerfectStart Green qPCR SuperMix (TransGen Biotech, AQ601) on a LightCycler 96 Detection System (Roche) using GAPDH for normalization. Primers used in this study are listed in Supplementary Table S4.

### DNA constructs

The SALIS expressing construct was purchased from YouBio Biological Technology Co., Ltd. (http://www.youbio.cn) with sequencing verification.

### Immunoblotting and IHC assays

Total cell extracts were prepared and assayed by western blot as previously described [48]. The following primary antibodies and dilutions were used: IGFBP3 (Santa Cruz Biotechnology, sc-374365, 1:2000), STAT5A (Santa Cruz Biotechnology, sc-271542, 1:2000), BAX (Santa Cruz Biotechnology, sc-7480, 1:2000), BCL-2 (Abcam, ab59348, 1:2000), Caspase-3 (Santa Cruz Biotechnology, sc-56053, 1:2000), Caspase-7 (Santa Cruz Biotechnology, sc-56063, 1:2000), and GAPDH (Santa Cruz Biotechnology, sc-25778,1:5000). The following secondary antibodies were also used: anti-mouse IgG-horseradish peroxidase (HRP), anti-rabbit IgG-HRP, and anti-goat IgG-HRP (Santa Cruz Biotechnology). Bound antibodies were visualized with the Luminata Forte Western HRP substrate (Millipore).

Xenograft tumors were formalin-fixed and paraffin-embedded and sectioned for IHC staining. The following antibodies were used: IGFBP3 (Santa Cruz Biotechnology, sc-374365, 1:100), Caspase-7 (Santa Cruz Biotechnology, sc-56063, 1:100). Stained sections were imaged using BX53 microscope (Olympus) to get representative images for statistical analysis.

### In situ hybridization (ISH) and Fluorescence in situ hybridization (FISH)

Antisense single-stranded DNA probe (Supplementary Table S4) was synthesized and end-labeled with digoxigenin (DIG) (Roche). ISH or FISH was performed in formalin-fixed paraffin-embedded HCC sections or slides cultured with cultured HCC cells. The pre-hybridization, hybridization, anti-DIG-HRP IgG fraction monoclonal (Jackson, 200-032-156) incubation (1:200) and stained with DAB (Servicebio, G1211) was performed as described in previous studies [7, 26, 49]. Stained ISH or FISH sections were imaged with a ZEISS Axio Vert.A1 microscope and at least 10 representative images were collected for statistical analysis. The ISH or FISH staining was performed “blind” with respect to the different treatments.

### RNA pulldown

RNA pulldown was performed as previous described [7, 26, 49]. Biotinylated SALIS transcript and EGFP RNAs were transcribed using a MAXIscript T7/T3 in vitro transcription kit (Ambion) and Biotin RNA labeling Mix (Roche).

### RNA immunoprecipitation (RIP) assay

RIP was performed as described [49]. 5 μg antibodies against STAT5A (Santa Cruz Biotechnology, sc-271542) or isotype IgG (Santa Cruz Biotechnology, sc-2025) as a negative control was used.

### High-performance liquid chromatography-mass spectrometry (HPLC-MS) analysis

A 20 μg sample of immunoprecipitated protein mix was separated by sodium dodecyl sulfate-polyacrylamide gel electrophoresis (SDS-PAGE) and stained with Coomassie brilliant blue R250 and then processed with the Trypsin Profile IGD Kit (Sigma, PP0100). The resulting digest was processed with ZipTip C18 (Merck Millipore, ZTC18S096) and then subjected to analysis by Thermo Fisher Scientific orbitrap fusion LC-MS/MS in positive ion, linear, delayed-extraction mode. Calibration was carried out using a standard peptide mixture. The mass spectra were subjected to sequence database for searching with Proteome Discoverer v2.1 software (Thermo Scientific). The results of HPLC-MS are shown in Supplementary Table S3.

### Chromatin Isolation by RNA Purification (ChIRP)

Anti-sense DNA tiling probes for the selective retrieval of SALIS were designed through online designer (https://www.biosearchtech.com/products/rna-fish/chirp-probe-sets) and synthesized by Sangon Biotech (Shanghai) Co., Ltd. with BiotinTEG at the 3-prime end. The ChIRP assay was performed according to the guidance [50] and as previous described [7]. Retrieved SALIS RNA and DNA fragments expected to bind with SALIS were quantified by qRT-PCR.

### Xenograft mouse model

Briefly, 2.0 × 10^6^ cells were subcutaneously implanted into the left and right flanks of female athymic nude mice (BALB/C-nu/nu, 4–5 weeks old). The experiments were performed “blind” with respect to different treatments. Oligos or plasmids were prepared by pre-incubating 3 nM per mouse with EL Transfection or PL Transfection Reagent (TransGen Biotech) 15 min; injections were prepared using a final volume of 100 μl in serum-free DMEM. The tumor diameters were measured and recorded every two days to generate tumor growth curves. After tumor growth assessment, the tumors were excised and separated with snap-frozen for RNA and protein extraction or paraffin-embedded for IHC staining.

### Cell proliferation and colony forming assays

HCC cells (4000 per well) cultivated on 96-well plates were transfected with siRNA and cell proliferation was detected after 0, 24, 48, and 72 h using cell counting kit (TransGen Biotech, FP101) at 450 nm as described in the manual. For the colony forming assay, transfected cells were incubated in six-well plates at 1000 cells per well, which were maintained in DMEM and medium was replaced with twice. At day 7, plates were collected after being washed twice with PBS and fixed in 4% paraformaldehyde for 30 min. Finally, the cells were stained with 0.1% crystal violet. Visible colonies were photographed and counted.

### Flow cytometry assay

HCC cells were seeded on 35 mm dishes and transfected with siRNA and cultured for 36 h. TransDetect Annexin V-FITC/PI cell apoptosis detection kit (Multi sciences) was applied according to instructions. Cell death was detected and quantified using a Guava easyCyte Flow Cytometry System (Merk Millipore).

### Transwell assay

To assess cell migration, 2.0 × 10^5^ HepG2 cells transfected with siNC, siSALIS_01 and siSALIS_02 were seeded into the 8 μm upper chambers of 12-well plates (Merk Millipore) in serum-free DMEM. During culture at 37 °C for 24 h, the cells in the upper chambers were attracted by the culture medium in the lower chamber, through chemoattractant provided by the included 10% fetal bovine serum. The chambers were washed with PBS twice and fixed with 3.7% formaldehyde. Cells were permeabilized using 100% methanol at room temperature, stained with 0.1 crystal violet, and observed using a microscope after the cells remained in the wells being scraped off with cotton swabs.

### Matrigel invasiveness assay

For the assessment of invasive ability, Matrigel-coated chambers (Merck Millipore) were used to culture transfected HepG2 cells, of which 1.0 × 10^5^ cells were seeded into the upper chambers. Other treatments were performed as in the migration assay.

### Statistical analysis

Statistical tests were performed for independent-samples with an unpaired *t*-test or one-way ANOVA tests (SPSS version 17.0, SPSS Inc.). All statistical tests incorporated two-tailed tests and homogeneity of variance tests, and were considered to reflect significant differences if *P < 0.05, **P < 0.01, or ***P < 0.001. Details of statistical analyses including sample numbers (*n*) are included in the respective figure legends.

## Supplementary information

Supplementary Data

Supplementary Table S1

Supplementary Table S2

Supplementary Table S3

Supplementary Table S4

Original Data File

Checklist

## Data Availability

Mass spectrometry data have been deposited to ProteomeXchange (http://www.proteomexchange.org) with the dataset identifier PXD031844. RNA-seq data have been submitted in Gene Expression Omnibus (https://www.ncbi.nlm.nih.gov/geo/) with the accession code GSE196975. The original data of Western blots were all shown in Supplementary Original Data. All data generated or analyzed during this study are included in this published article and its Supplementary files and available from the corresponding authors on request.
