# Supplementary material for: SALIS transcriptionally represses IGFBP3/Caspase-7-mediated apoptosis by associating with STAT5A to promote hepatocellular carcinoma
Source: Cell Death Dis. 2022 Jul 23;13(7):642. doi: 10.1038/s41419-022-05094-z (PMC9308799; doi:10.1038/s41419-022-05094-z)
Supplement: Supplementary file 1 — Supplementary Data [file 41419_2022_5094_MOESM1_ESM.pdf]

Supplementary Figure S1

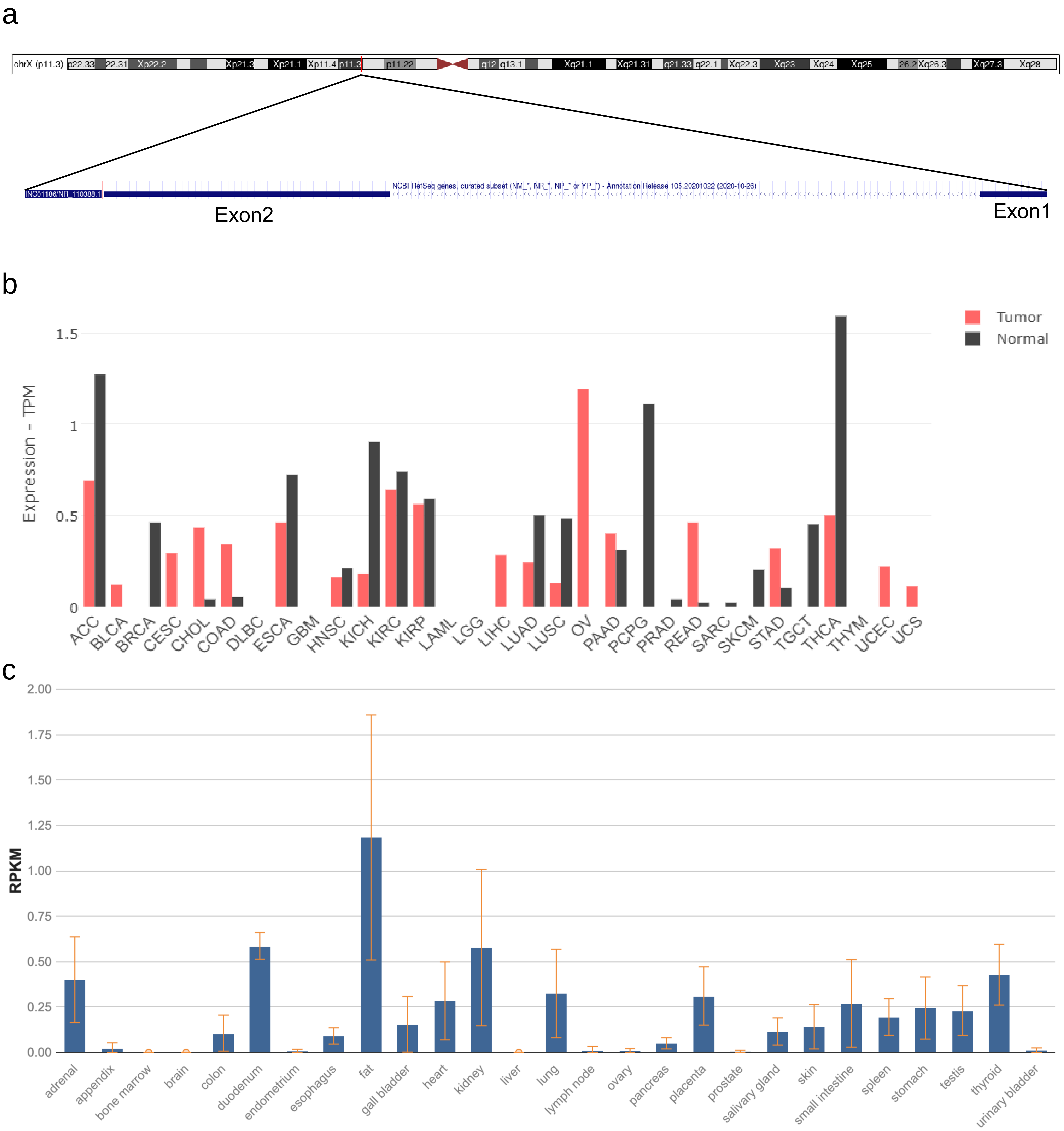

Supplementary Figure S1. (a) The gene structure and chromosomal location of SALIS. (b) Normalized SALIS expression levels in different types of cancer. The diagram showing the expression levels of SALIS in different types of cancer were analyzed and generated in Gene Expression Profiling Interactive Analysis (http://gepia.cancer-pku.cn/) using TCGA as data source. (c) The transcription mapping of SALIS expression in normal tissues from NCBI (https://www.ncbi.nlm.nih.gov/gene/?term=LINC01186).

Supplementary Figure S2

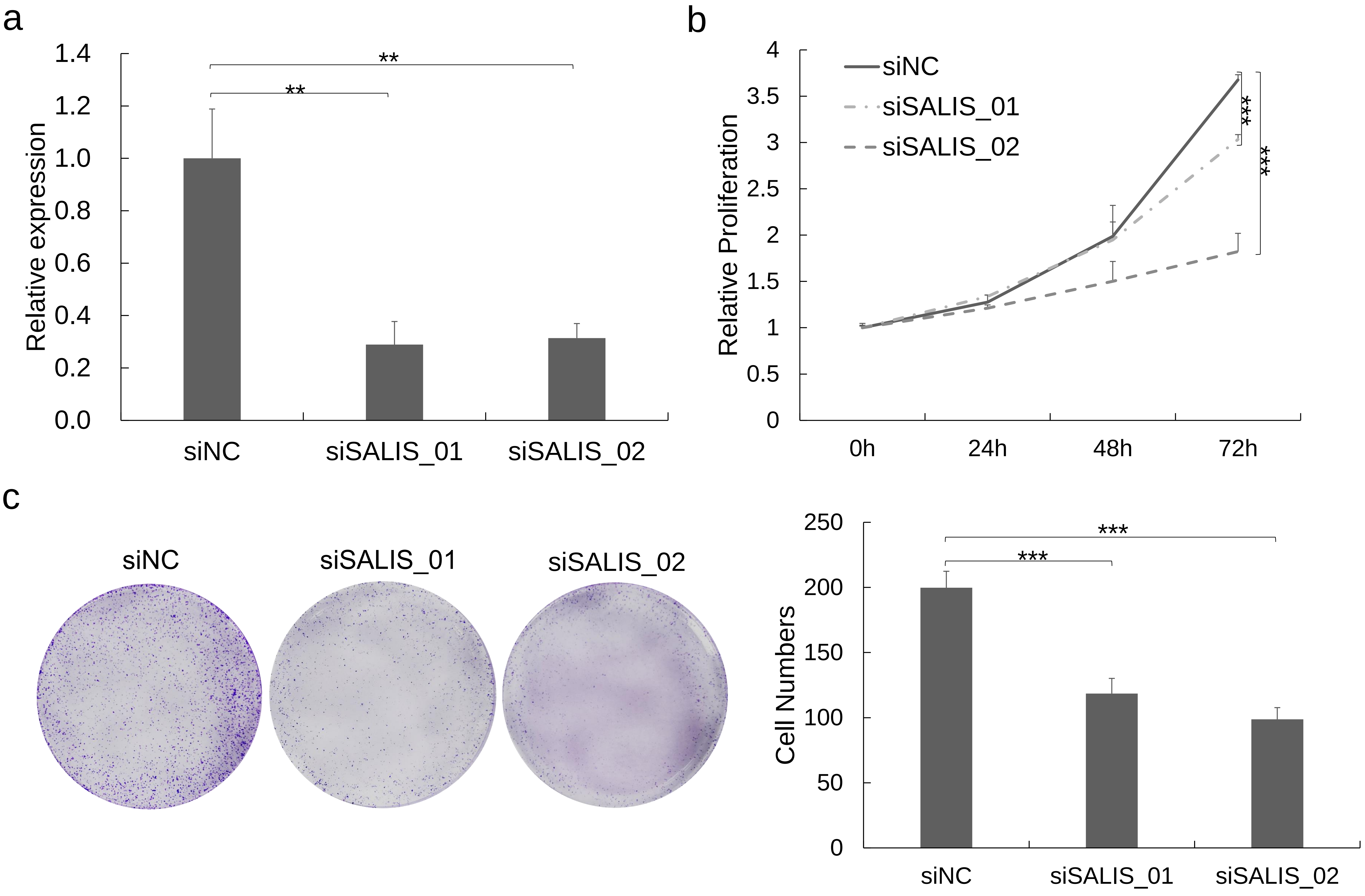

Supplementary Figure S2 (a) SALIS RNA expression was detected by qPCR after knockdown of SALIS by siRNAs in HCCLM3 cells. (b) Measurement of cell proliferation by CCK-8 assay in HCCLM3 cells treated with siSALIS oligos. (c) Colony formation assay were performed in HCCLM3 cells treated with siSALIS oligos. One-Way ANOVA or Dunnett’s multiple comparison test was performed to detect the significant difference. Means ± s.d. **P* < 0.05, ***P* < 0.01, ****P* < 0.001.

Supplementary Figure S3

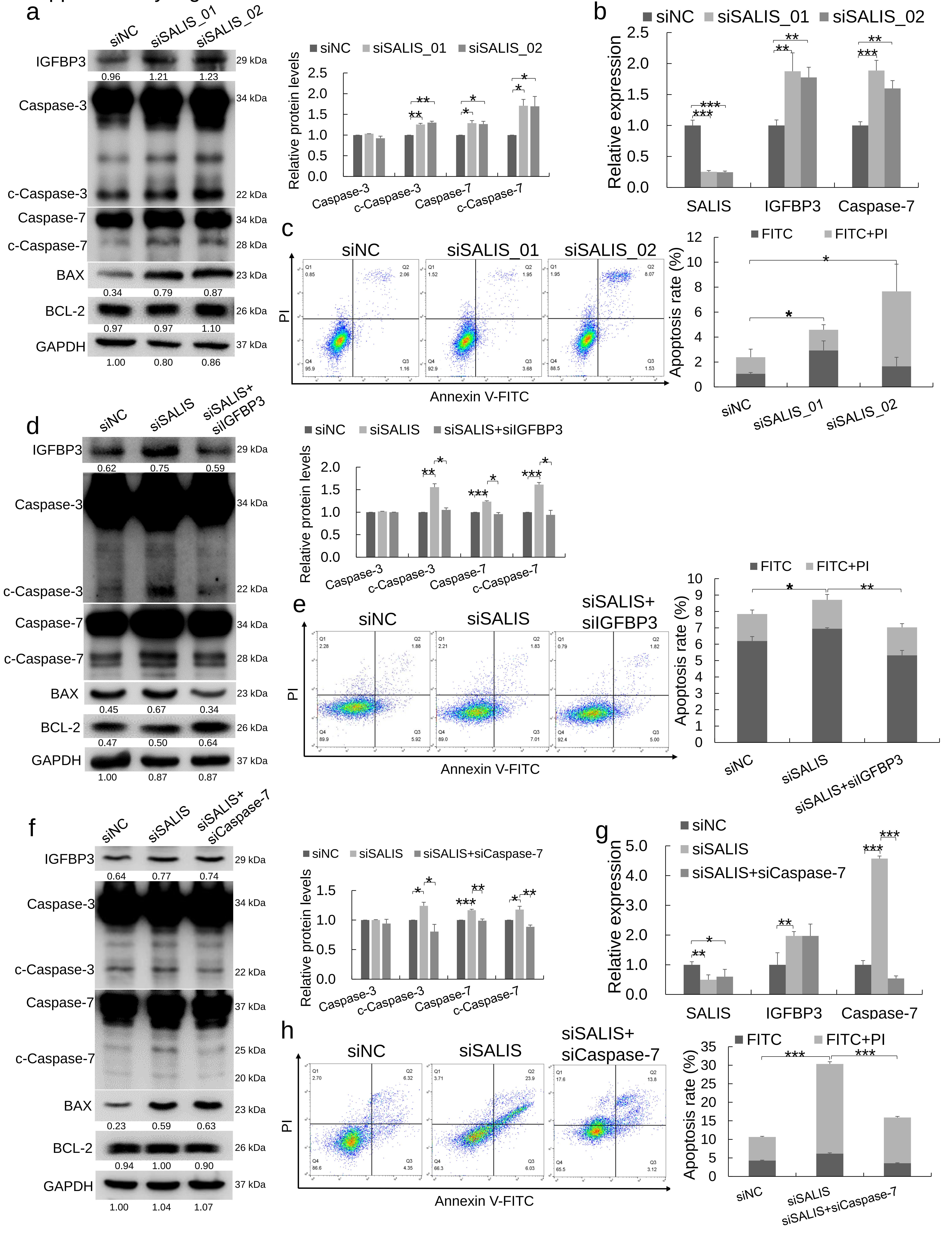

Supplementary Figure S3. (a) IGFBP3, BAX, BCL-2, Caspase-3 and Caspase-7 proteins were detected in HCCLM3 cells by Western blot after SALIS knockdown, GAPDH was using as loading control. The bar figure in right panel shows the statistically-analyzed relative protein levels of Caspase-3, Caspase-7 and their cleaved forms. (b) IGFBP3 and Caspase-7 RNA expression was detected by qPCR after knockdown of SALIS by siRNAs in HCCLM3 cells. (c) Apoptosis was detected by flow cytometry with Annexin V/PI double staining in HCCLM3 cells after depletion of SALIS. The bar figure in lower panel shows the statistically-analyzed apoptosis rate. (d) Western blot detection of IGFBP3, BAX, BCL-2, Caspase-3 and Caspase-7 in HCCLM3 cells treated with siNC, siSALIS or siSALIS+siIGFBP3. The bar figure in right panel shows the statistically-analyzed relative protein levels of Caspase-3, Caspase-7 and their cleaved forms. (e) Apoptosis was detected by flow cytometry with Annexin V/PI double staining in HCCLM3 cells with siNC, siSALIS or siSALIS+siIGFBP3. (f) IGFBP3, BAX, BCL-2, Caspase-3 and Caspase-7 proteins were detected in siNC, siSALIS or siSALIS+siCaspase-7 treated HepG2 cells by Western blot, GAPDH was using as loading control. The bar figure in right panel shows the statistically-analyzed relative protein levels of Caspase-3, Caspase-7 and their cleaved forms. (g) IGFBP3 and Caspase-7 RNA expression was detected by qPCR after knockdown of SALIS or both SALIS and Caspase-7 by siRNAs in HepG2 cells. (h) Apoptosis was detected by flow cytometry with Annexin V/PI double staining in siSALIS and siSALIS+siCaspase-7 treated HepG2 cells. The bar figure in right panel shows the statistically-analyzed apoptosis rate. The bar figure in lower panel shows the statistically-analyzed apoptosis rate. One-Way ANOVA or Dunnett’s multiple comparison test was performed to detect the significant difference. Means ± s.d. $^{*}P < 0.05$, $^{**}P < 0.01$, $^{***}P < 0.001$.

Supplementary Figure S4

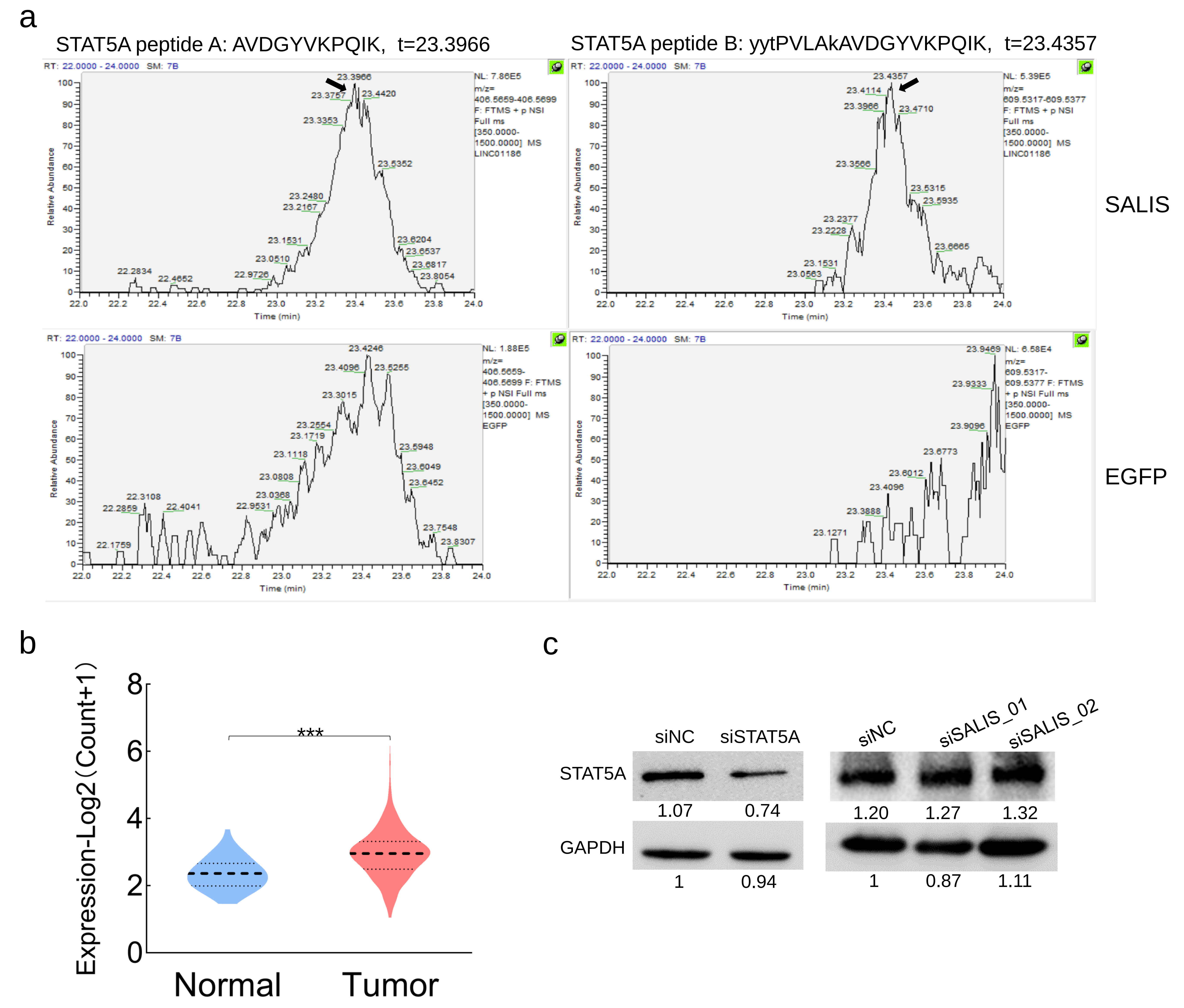

Supplementary Figure S4. (a) Two peptides of STAT5A identified by HPLC-MS. The chromatographic analysis of the protein mixes precipitated by SALIS RNA transcript or control EGFP RNA. Black arrows indicate the identified STAT5A peptide peaks in the SALIS-pulldown sample, which were lacking in control EGFP sample. (b) The expression of STAT5A in HCC (374 cases) compared with normal tissues (50 cases). The original expression data is from The Cancer Genome Atlas Program (TCGA) database. (c) The protein levels of STAT5A in HepG2 cells treated with siNC, siSTAT5A or siSALIS.

Supplementary Figure S5

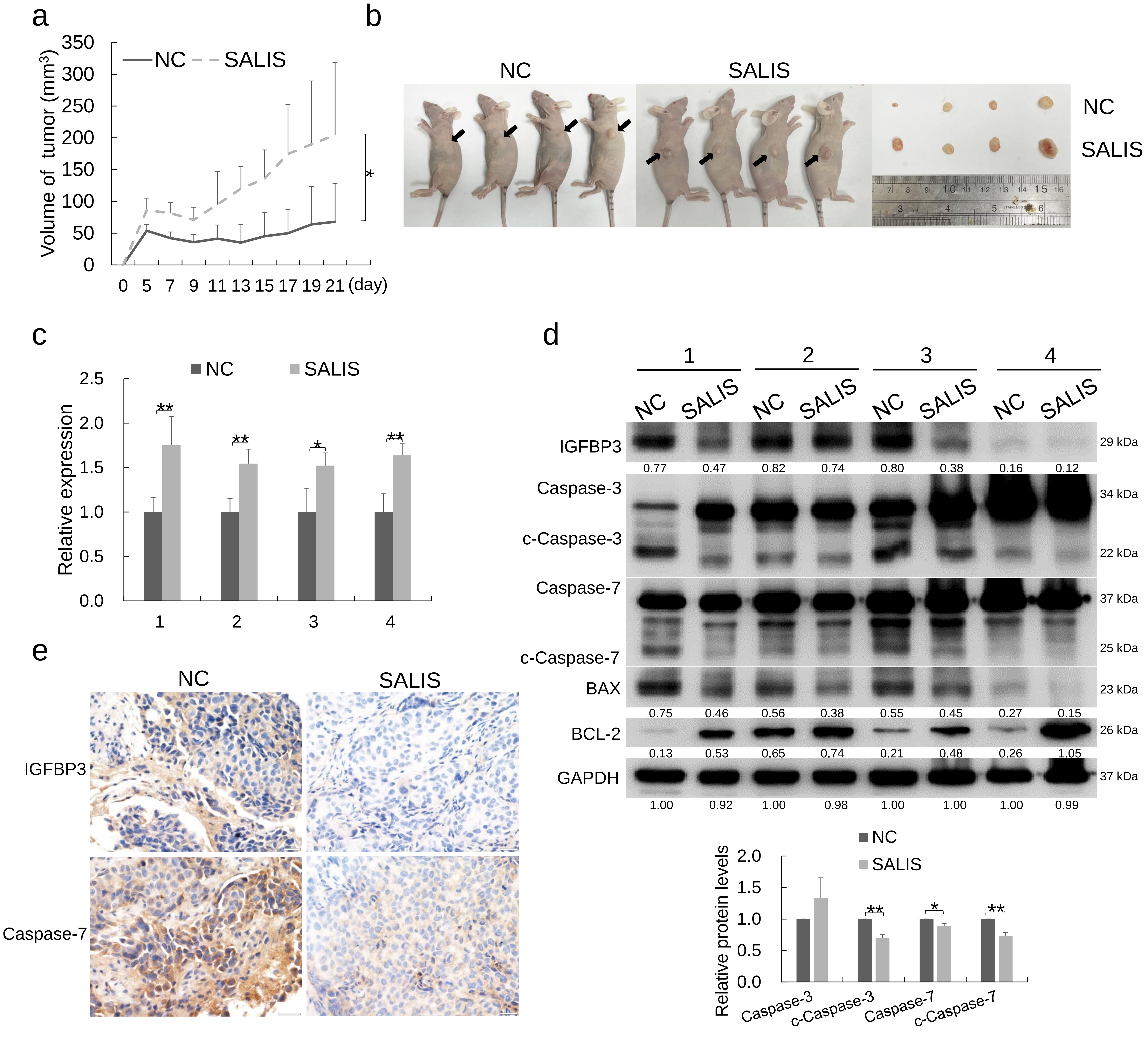

Supplementary Figure S5. (a) SALIS overexpression promotes tumor growth in mice xenograft model. Tumor volumes (mm^3) were plotted according to day. (b) The mice were sacrificed at the end of the experiment. The left two graph show the mice harboring xenograft tumors. Black arrows indicate the xenografts. The right graph shows the dissected tumors from the experimental mice. (c) SALIS expression was detected in the dissected xenograft tumors by qPCR. (d) The protein levels of IGFBP3, BAX, BCL-2, Caspase-3 and Caspase-7 were detected in SALIS overexpression groups together with control groups by Western blot. GAPDH was used as loading control. The bar figure in lower panel shows the statistically-analyzed relative protein levels of Caspase-3, Caspase-7 and their cleaved forms. (e) Immunohistochemical (IHC) staining of IGFBP3 and Caspase-7 expression of the xenograft tumors. The data are expressed as means ± s.d., n=4. Scale bar: 20 μM. *$P < 0.05$, **$P < 0.01$, ***$P < 0.001$.

Supplementary Figure S6

HepG2 cell line authentication

样品数量： 1

样品性状： 细胞系

检测项目： STR

送检单位： **富衡**

检测方法： 用 Axygen 的基因组抽提试剂盒提取 DNA，采用 21- STR 扩增方案扩增，在 ABI 3730XL 型遗传分析仪上对 STR 位点和性别基因 Amelogenin 进行检测。

检 测 结 果

(一) 检验基本情况

公司编号	多等位基因	匹配细胞系	细胞库	EV 值	匹配说明
20181221-02	无	HEPG2/C3A	DSMZ	1.0	完全匹配

样本基因型检验结果

- 多等位基因指三等位及以上基因现象。
- 本次检测各细胞分型结果良好。

(二) 各样本描述

- 20181221-02：该株细胞 DNA 分型在细胞系检索中找到**完全匹配**的细胞系，ATCC 数据库显示细胞名为 **HepG2/C3A**，细胞号对应 **CRL-10741**。本次检测在该细胞系中**没有发现多等位基因**。

备注： 待测细胞系与收录于 ATCC, DSMZ, JCRB 和 RIKEN 数据库的细胞系 STR 数据进行比对，未收录于以上细胞库的细胞系将无法匹配。

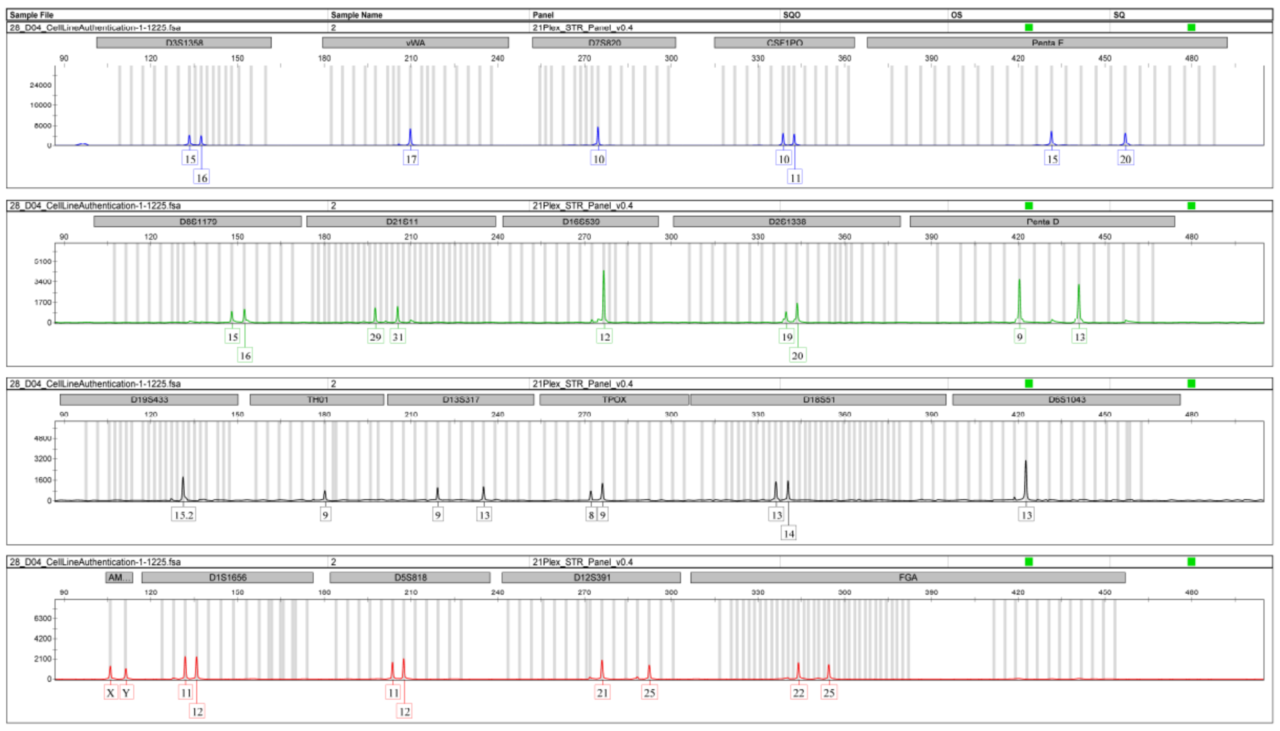

(三) 样本分型结果

细胞的 STR 位点和 Amelogenin 位点的基因分型结果						
Loci	送检细胞 STR 信息			细胞库细胞 STR 信息		
	送检细胞名：2			细胞库细胞名：HepG2/C3A		
	Allele1	Allele2	Allele3	Allele1	Allele2	Allele3
D5S818	11	12		11	12	
D13S317	9	13		9	13	
D7S820	10	10		10	10	
D16S539	12	12		12	12	
VWA	17	17		17	17	
TH01	9	9		9	9	
AMEL	X	Y		X	Y	
TPOX	8	9		8	9	
CSF1PO	10	11		10	11	
D12S391	21	25				
FGA	22	25				
D2S1338	19	20				
D21S11	29	31				
D18S51	13	14				
D8S1179	15	16				
D3S1358	15	16				
D6S1043	13	13				
PENTAE	15	20				
D19S433	15.2	15.2				
PENTAD	9	13				
D1S1656	11	12				

其 他 说 明

(一) 分型方案及位点分布

	方案 1	方案 2	方案 3	方案 4
1	D3S1358	D8S1179	D19S433	AMEL
2	VWA	D21S11	TH01	D1S1656
3	D7S820	D16S539	D13S317	D5S818
4	CSF1PO	D2S1338	TPOX	D12S391
5	PENTAE	PENTAD	D18S51	FGA
6			D6S1043	

实验方案及位点

(二) STR 数据库比对

本公司采用 DSMZ tools 进行细胞系比对，其中包含来自于 ATCC, DSMZ, JCRB 和 RIKEN 数据库的 2455 个细胞系 STR 数据。如果待检测细胞未收录于以上细胞库或这是自行建立的新细胞系将无法进行比对，用户需根据细胞分型结果自行与其他数据库进行比对。

签发日期：2018-12-29

Supplementary Figure S7

HCCLM3 cell line authentication

样品数量： 1

样品性状： 细胞系

检测项目： STR

送检单位： 富衡

检测方法： 用 Axygen 的基因组抽提试剂盒提取 DNA，采用 20- STR 扩增方案扩增，在 ABI 3730XL 型遗传分析仪上对 STR 位点和性别基因 Amelogenin 进行检测。

检 测 结 果

(一) 检验基本情况

	多等位基因	匹配细胞系	细胞库	EV 值	匹配说明
20180503-02	无		DSMZ		无匹配

样本基因型检验结果

- 多等位基因指三等位及以上基因现象。
- 本次检测各细胞分型结果良好。

(二) 各样本描述

- 20180503-02：该株细胞 DNA 分型在细胞系检索中**没有找到匹配**的细胞系，本次检测在该细胞系中**没有发现多等位基因**。（该细胞系未发现多等位基因、未发现交叉污染，细胞系无异常，因数据库未收录 HCCLM3 细胞系相关 STR 数据信息无法匹配

备注： 待测细胞系与收录于 ATCC, DSMZ, JCRB 和 RIKEN 数据库的细胞系 STR 数据进行比对，未收录于以上细胞库的细胞系将无法匹配。

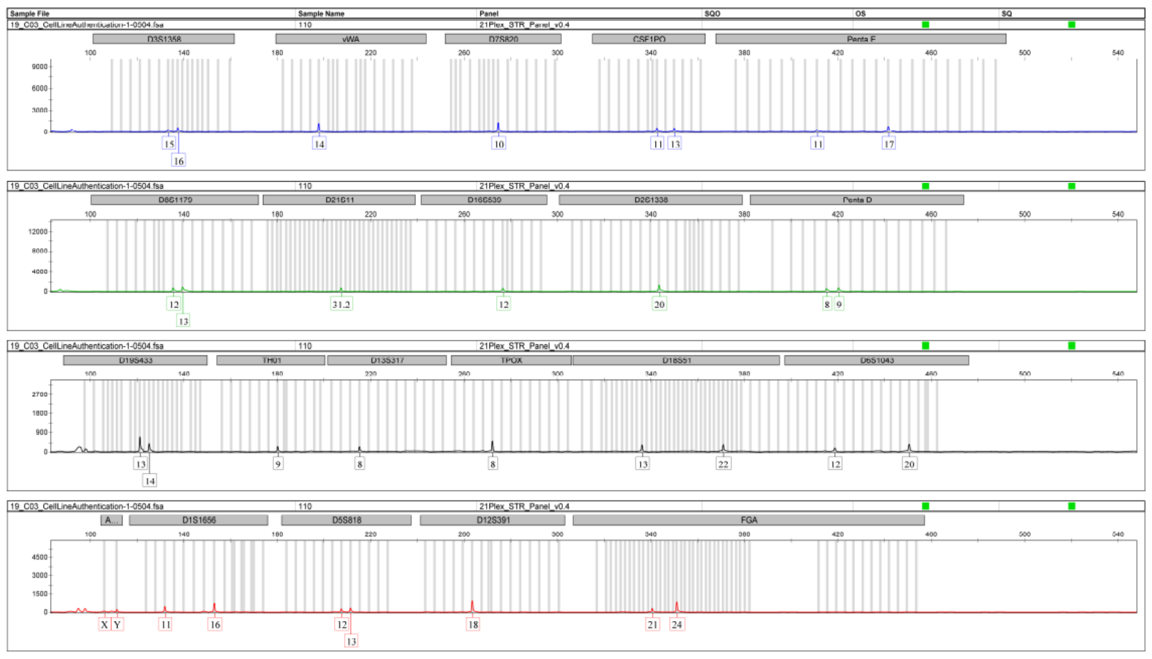

其 他 说 明

(一) 分型方案及位点分布

	方案 1	方案 2	方案 3	方案 4
1	TH01	TPOX	D3S1358	AMEL
2	D12S391	VWA	D13S317	D5S818
3	D7S820	D8S1179	D6S1043	D2S1338
4	CSF1PO	PENTAD	D16S539	D21S11
5	FGA		D19S433	D18S51
6	PENTAE			

实验方案及位点

(二) STR 数据库比对

本公司采用 DSMZ tools 进行细胞系比对，其中包含来自于 ATCC, DSMZ, JCRB 和 RIKEN 数据库的 2455 个细胞系 STR 数据。如果待检测细胞未收录于以上细胞库或这是自行建立的新细胞系将无法进行比对，用户需根据细胞分型结果自行与其他数据库进行比对。

签发日期： 2018 年 05 月 10 日

样本分型结果

细胞 20180503-02 的 STR 位点和 Amelogenin 位点的基因分型结果						
Loci	送检细胞 STR 信息			细胞库细胞 STR 信息		
	送检细胞名：110			细胞库细胞名：		
	Allele1	Allele2	Allele3	Allele1	Allele2	Allele3
D5S818	12	13				
D13S317	8	8				
D7S820	10	10				
D16S539	12	12				
VWA	14	14				
TH01	9	9				
AMEL	X	Y				
TPOX	8	8				
CSF1PO	11	13				
D12S391	18	18				
FGA	21	24				
D2S1338	20	20				
D21S11	31.2	31.2				
D18S51	13	22				
D8S1179	12	13				
D3S1358	15	16				
D6S1043	12	20				
PENTAE	11	17				
D19S433	13	14				
PENTAD	8	9				

细胞出库质检单

一、产品信息

1 细胞名称：HCCLM3

2 出库日期：2021/07/05

二、检测项目及结果

检测项目	检测结果	
生长特性	贴壁/悬浮	
细胞形态	上皮样	
细胞密度	>75%	
细胞纯度	---	
细胞总数	~1×10^6	
细胞活力	>95%	
[illegible]	有□	无☑
[illegible]	有□	无☑
[illegible]	有□	无☑
支原体	有□	无☑
[illegible]	有□	无☑
[illegible]	有□	无☑
[illegible]	有□	无☑

三、质检员及质检日期

质检员：[illegible]

质检日期：

2021.07.05

上海富衡生物科技有限公司 质检专用章

质检合格 [illegible]
