# Supplementary material for: SALIS transcriptionally represses IGFBP3/Caspase-7-mediated apoptosis by associating with STAT5A to promote hepatocellular carcinoma
Source: Cell Death Dis. 2022 Jul 23;13(7):642. doi: 10.1038/s41419-022-05094-z (PMC9308799; doi:10.1038/s41419-022-05094-z)

Original data of Western blots

Related to Figure 4a

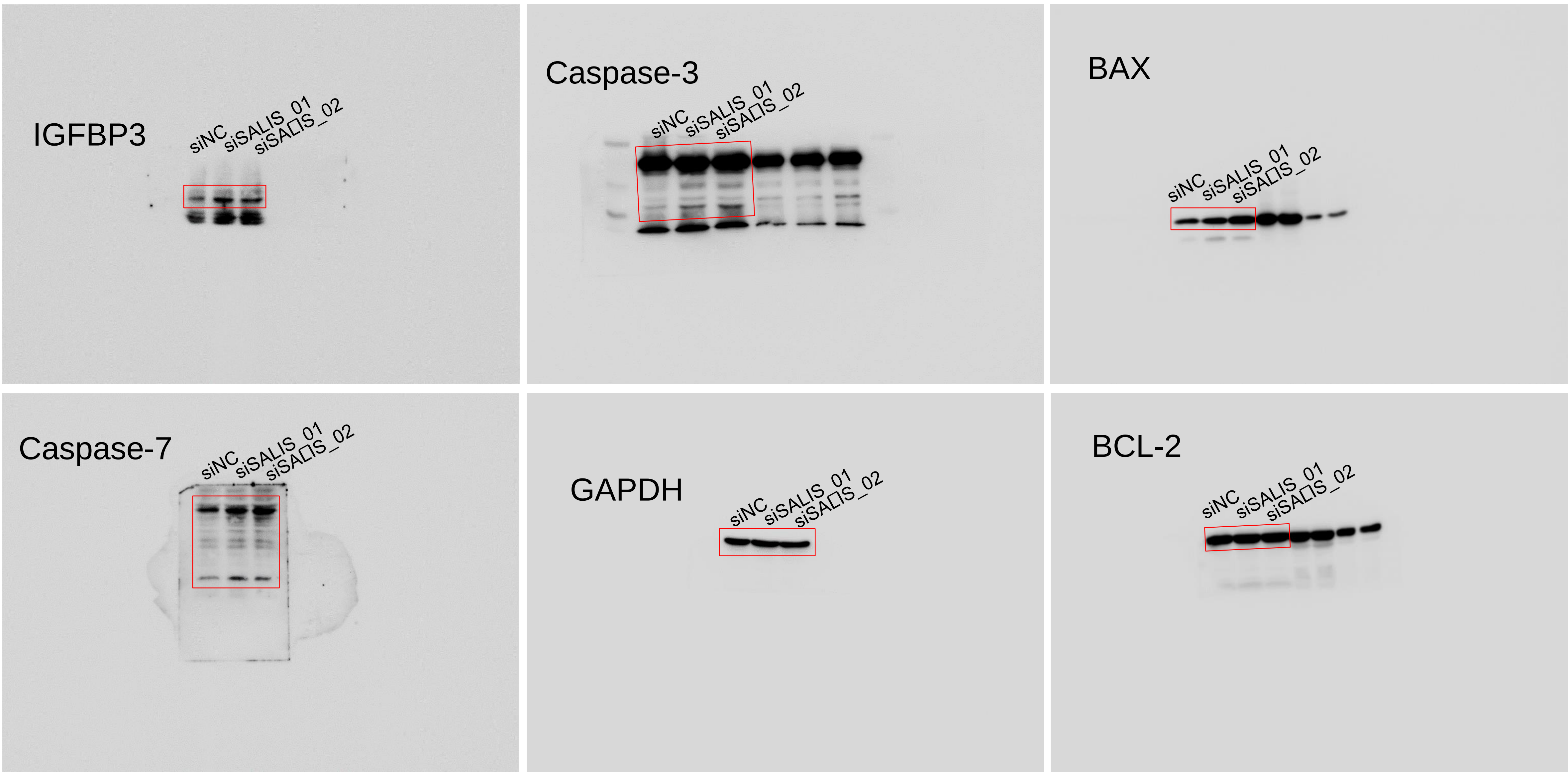

Related to Figure 4d

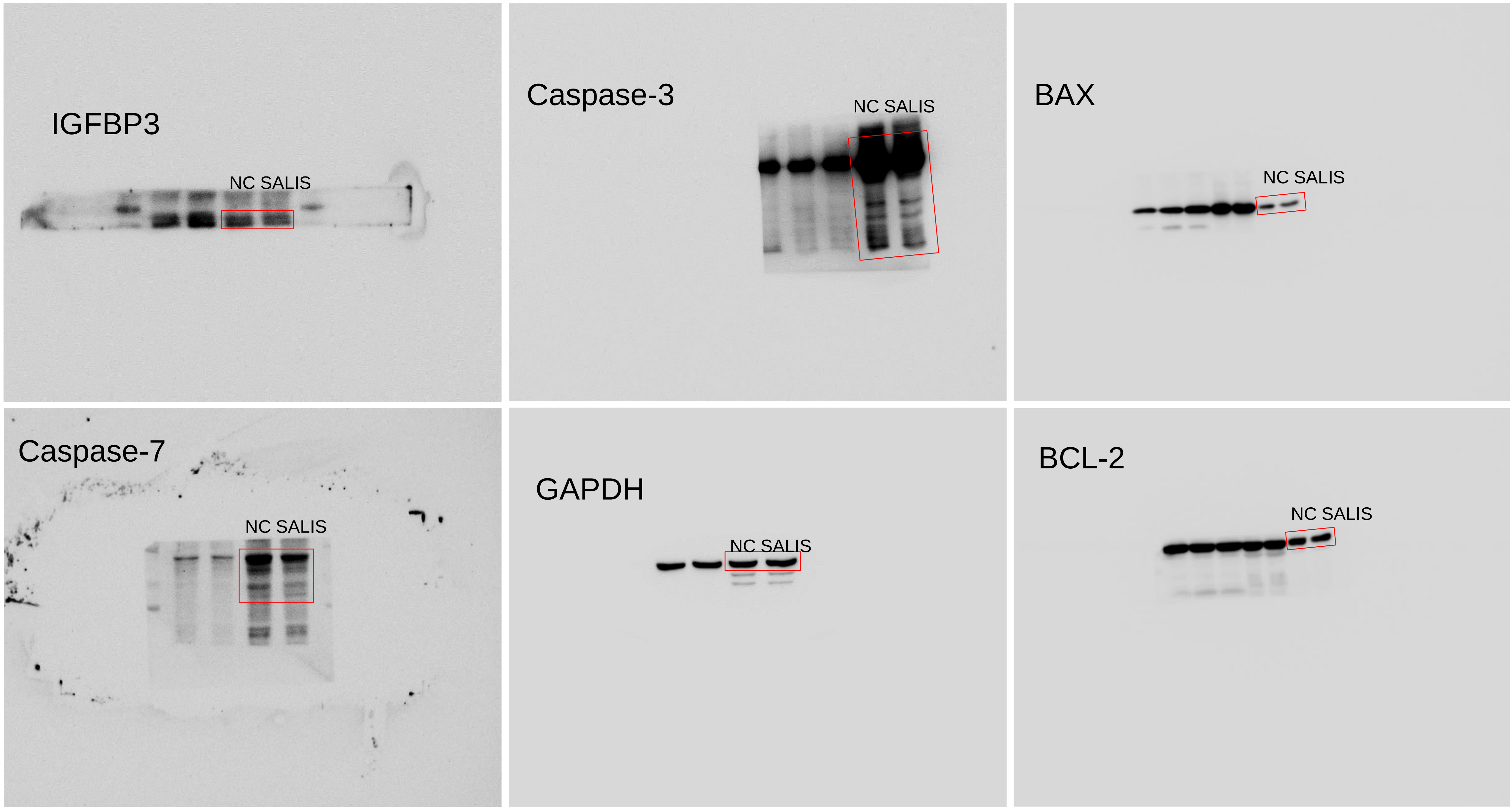

Related to Figure 4g

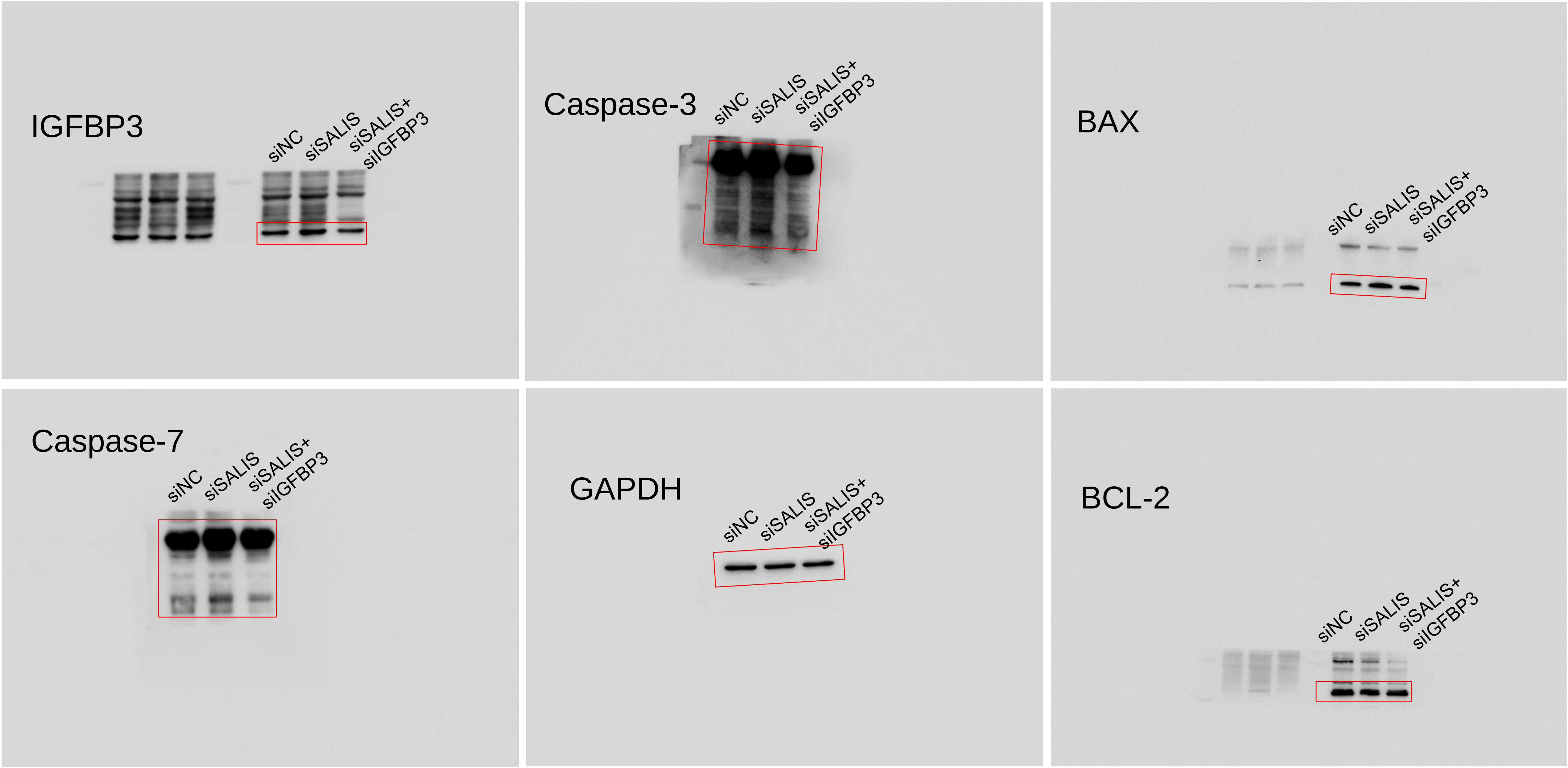

Related to Figure 5b

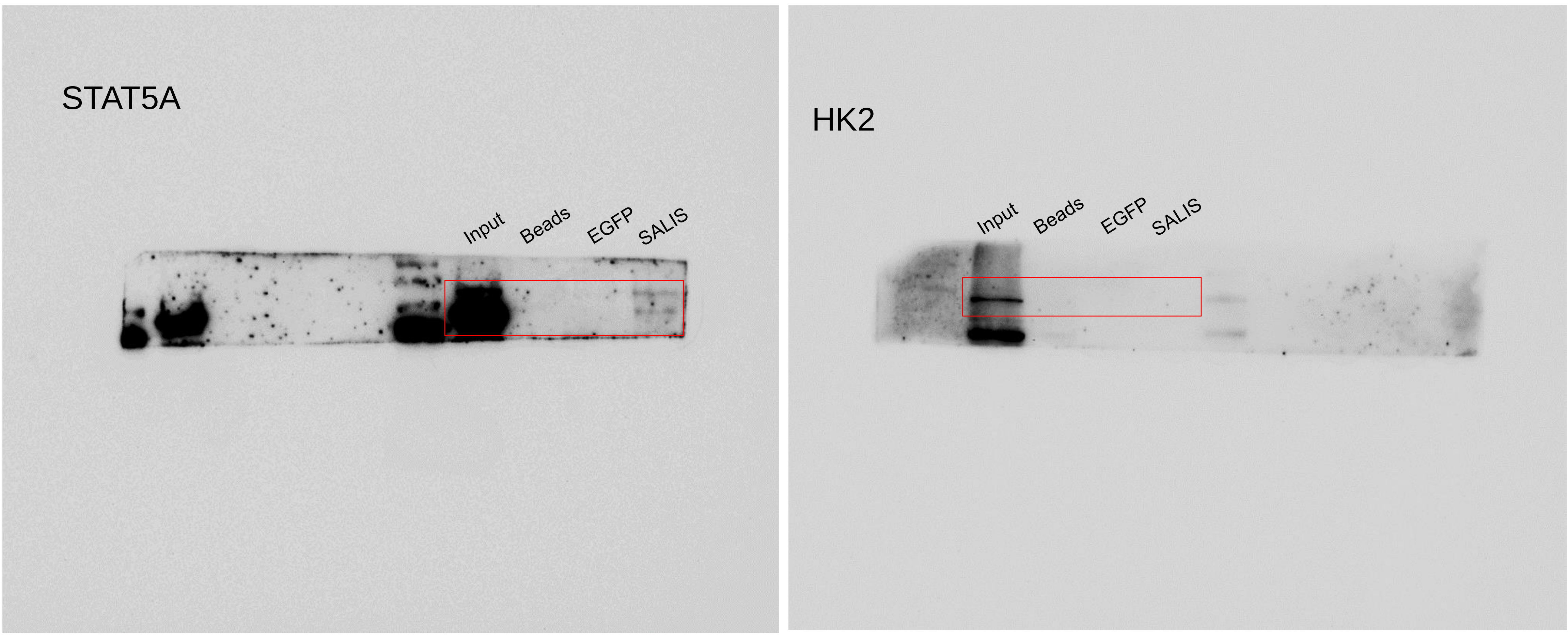

Related to Figure 6b

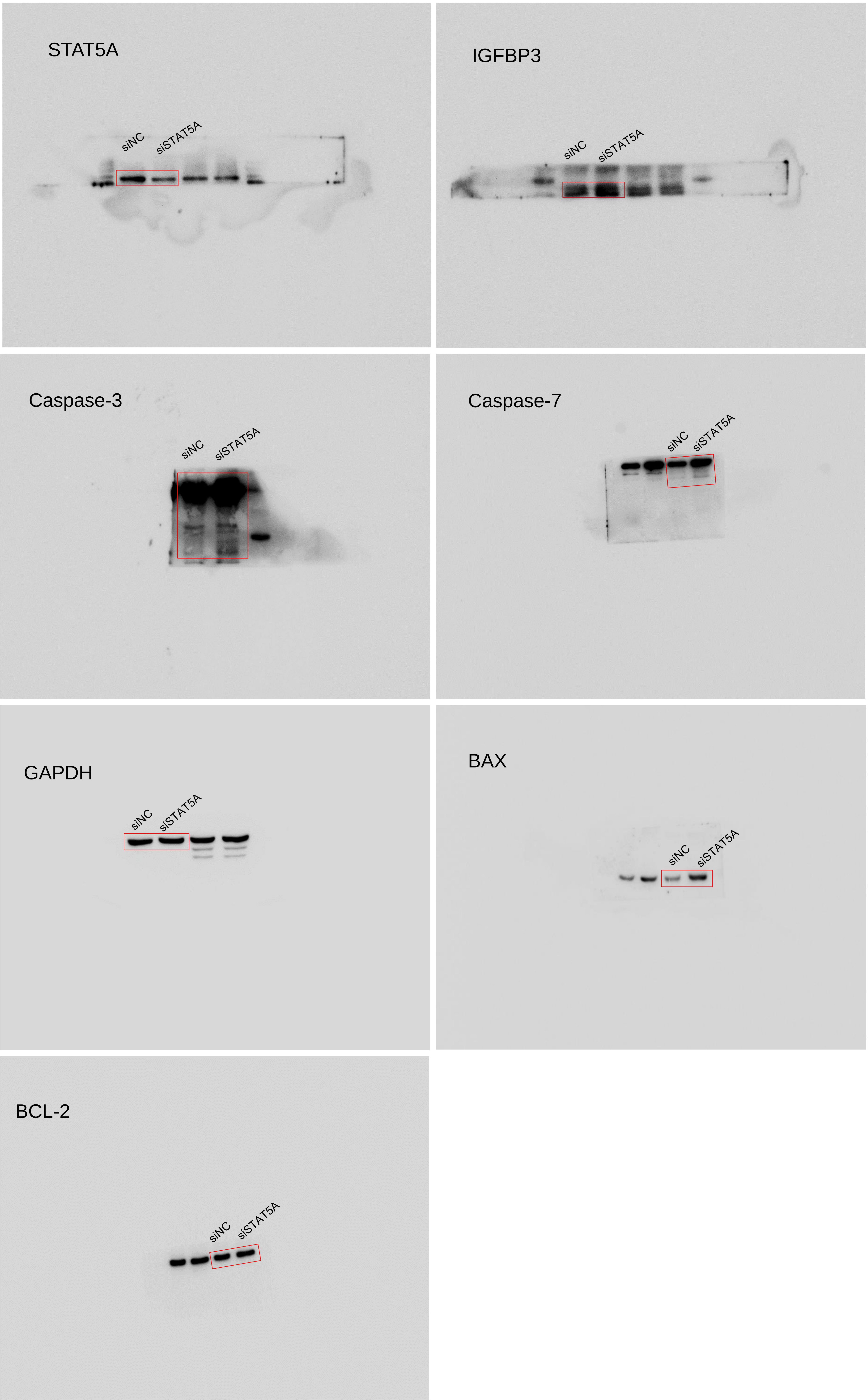

Related to Figure 6d

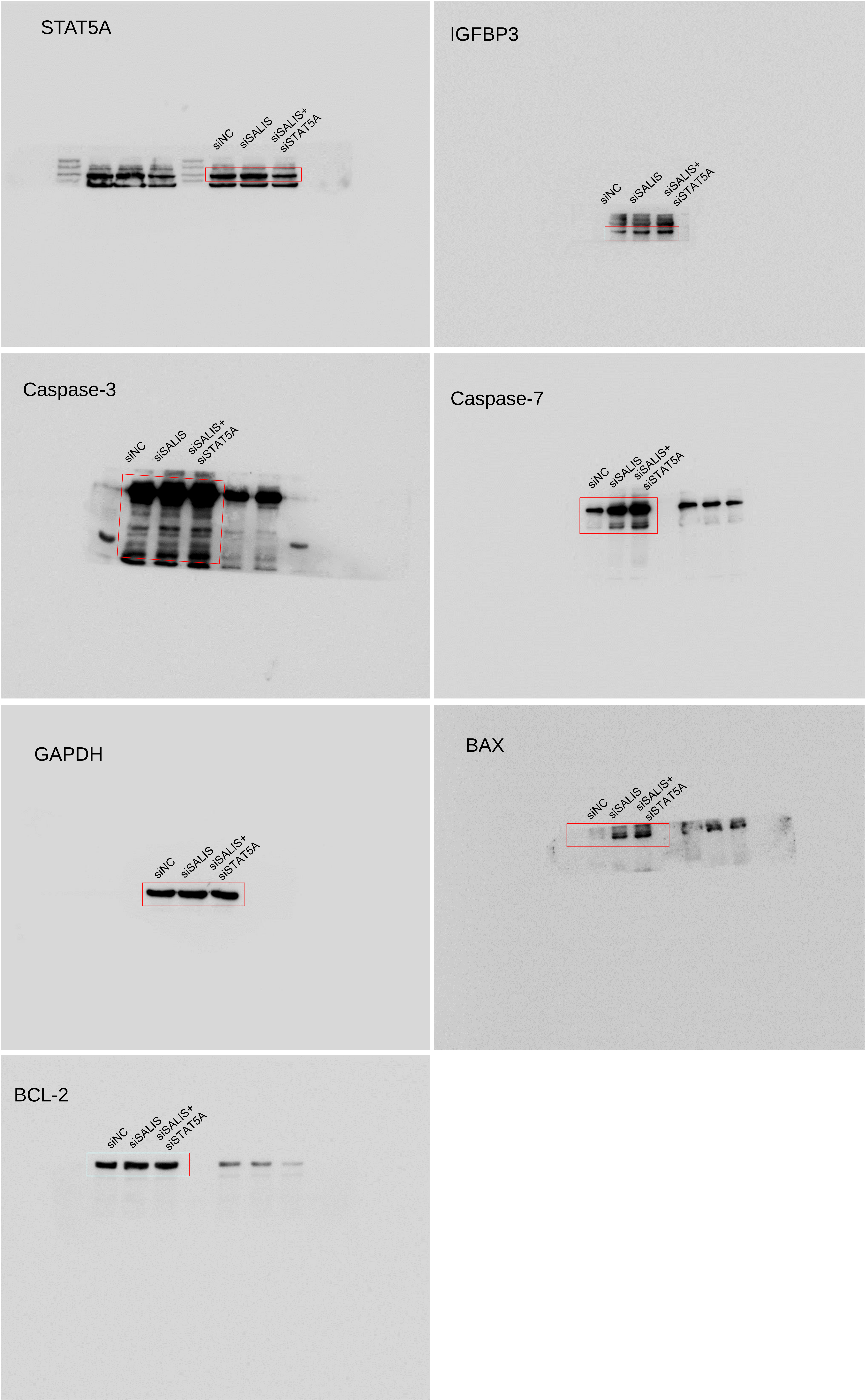

Related to Figure 7e

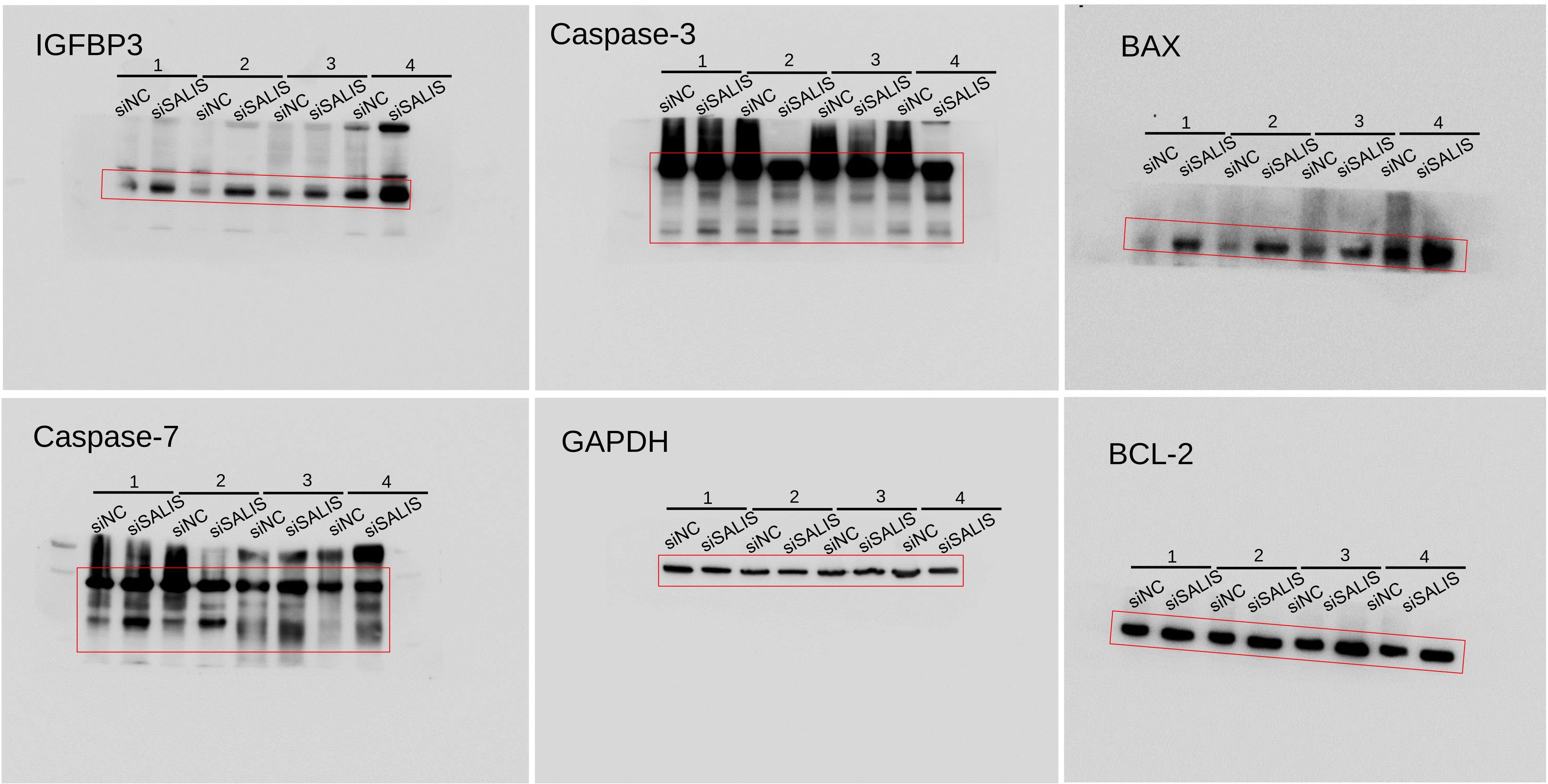

Related to Supplementary Figure S3a

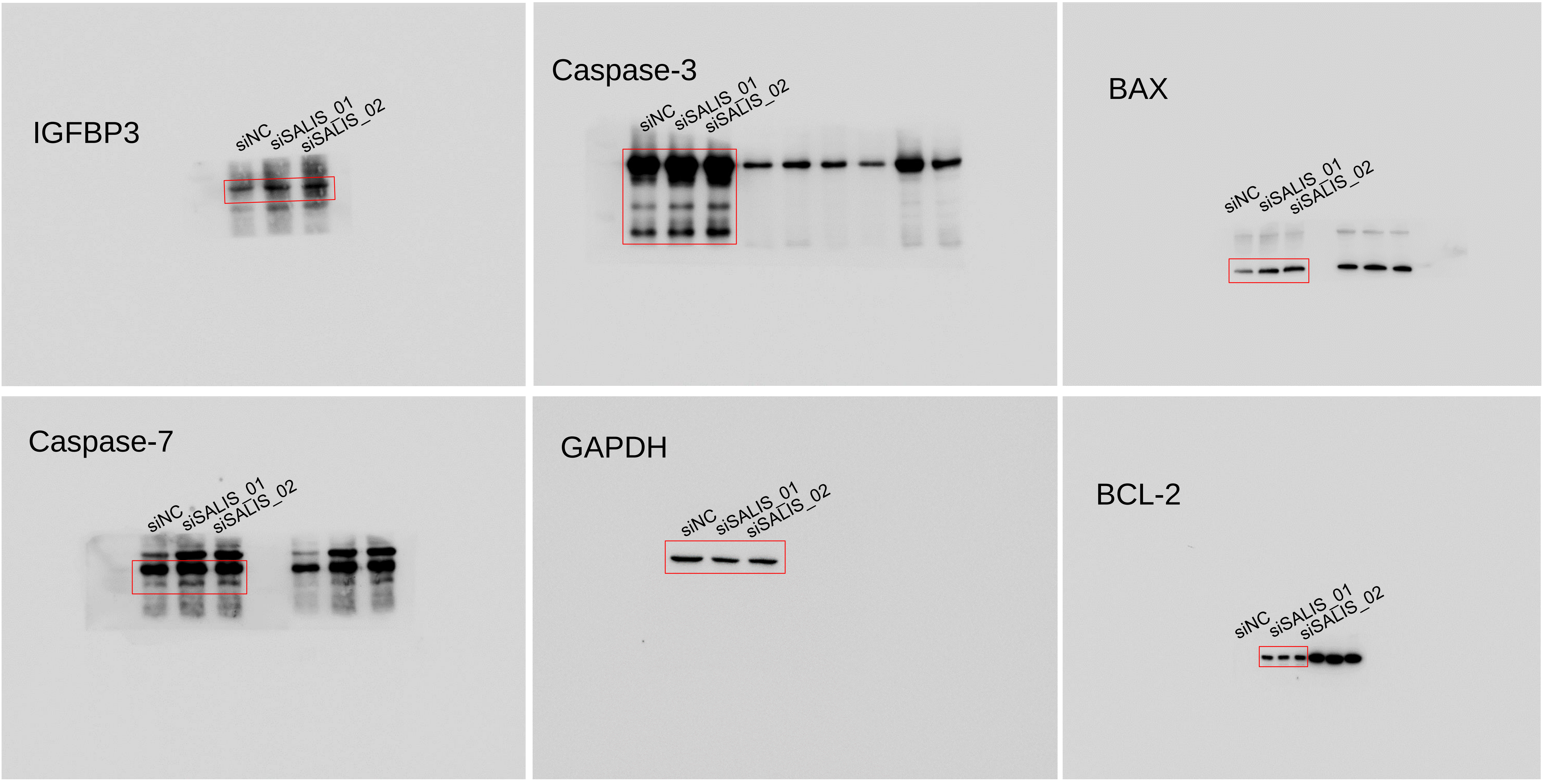

Related to Supplementary Figure S3d

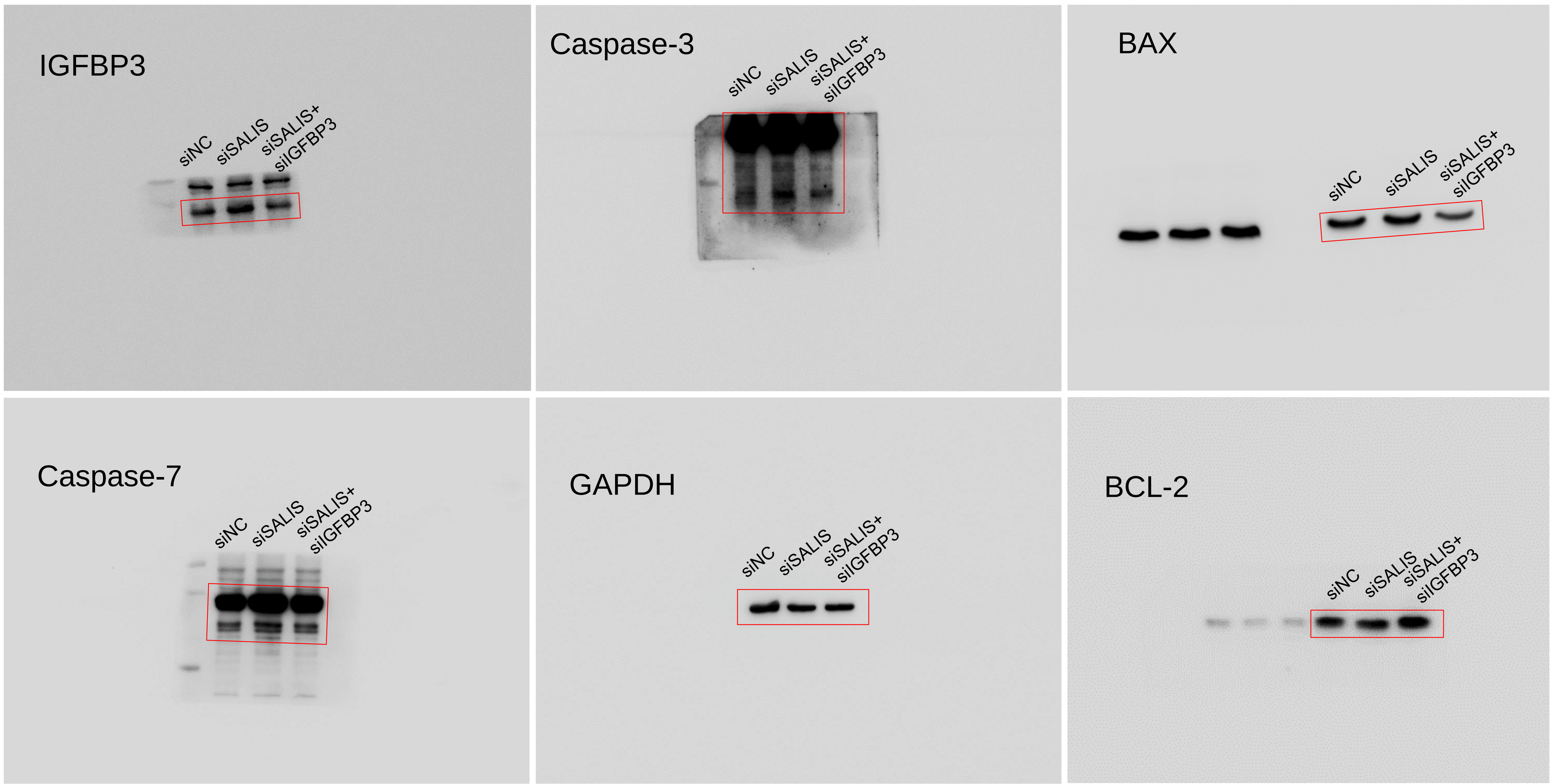

Related to Supplementary Figure S3f

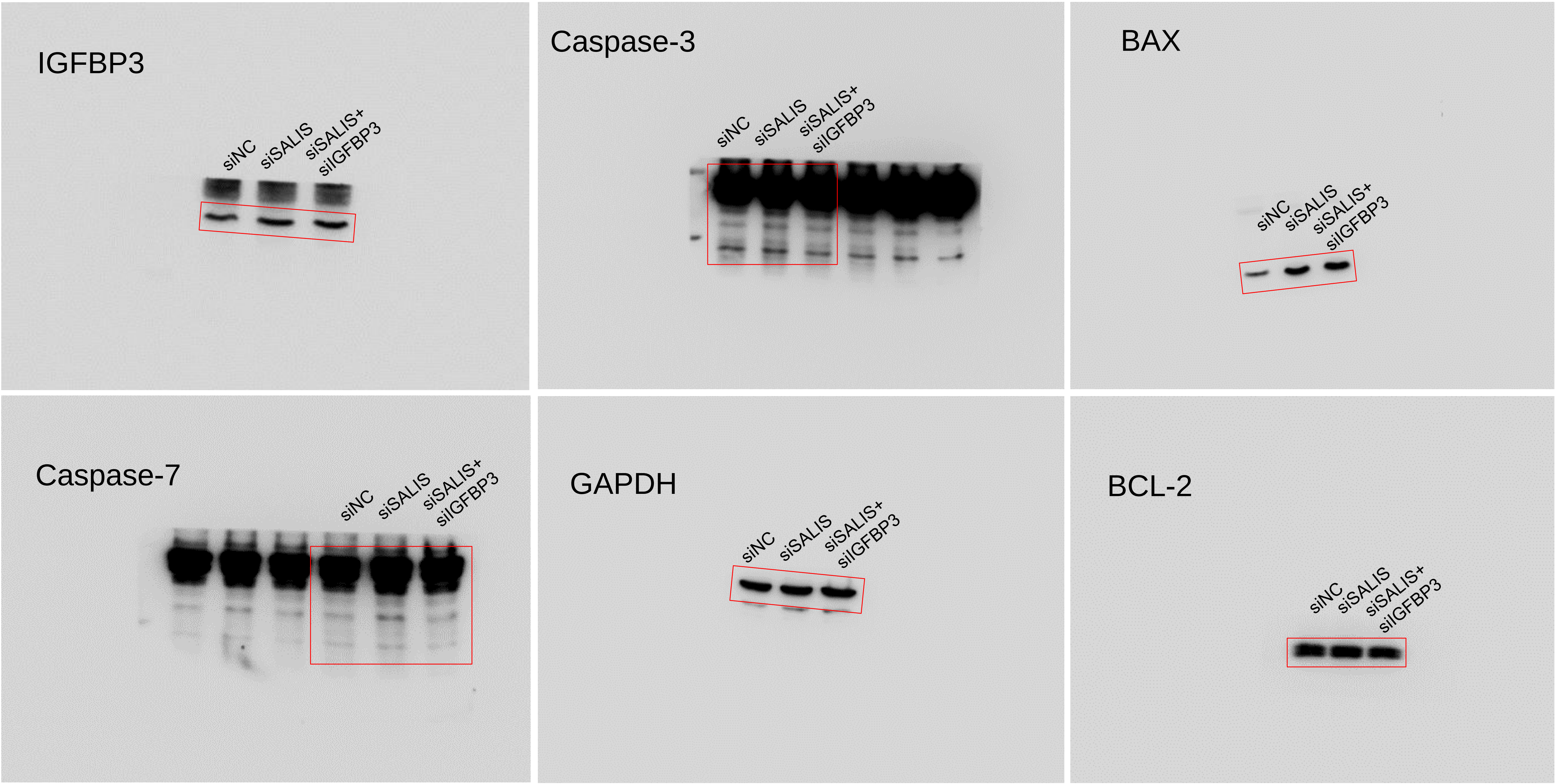

Related to Supplementary Figure S4b

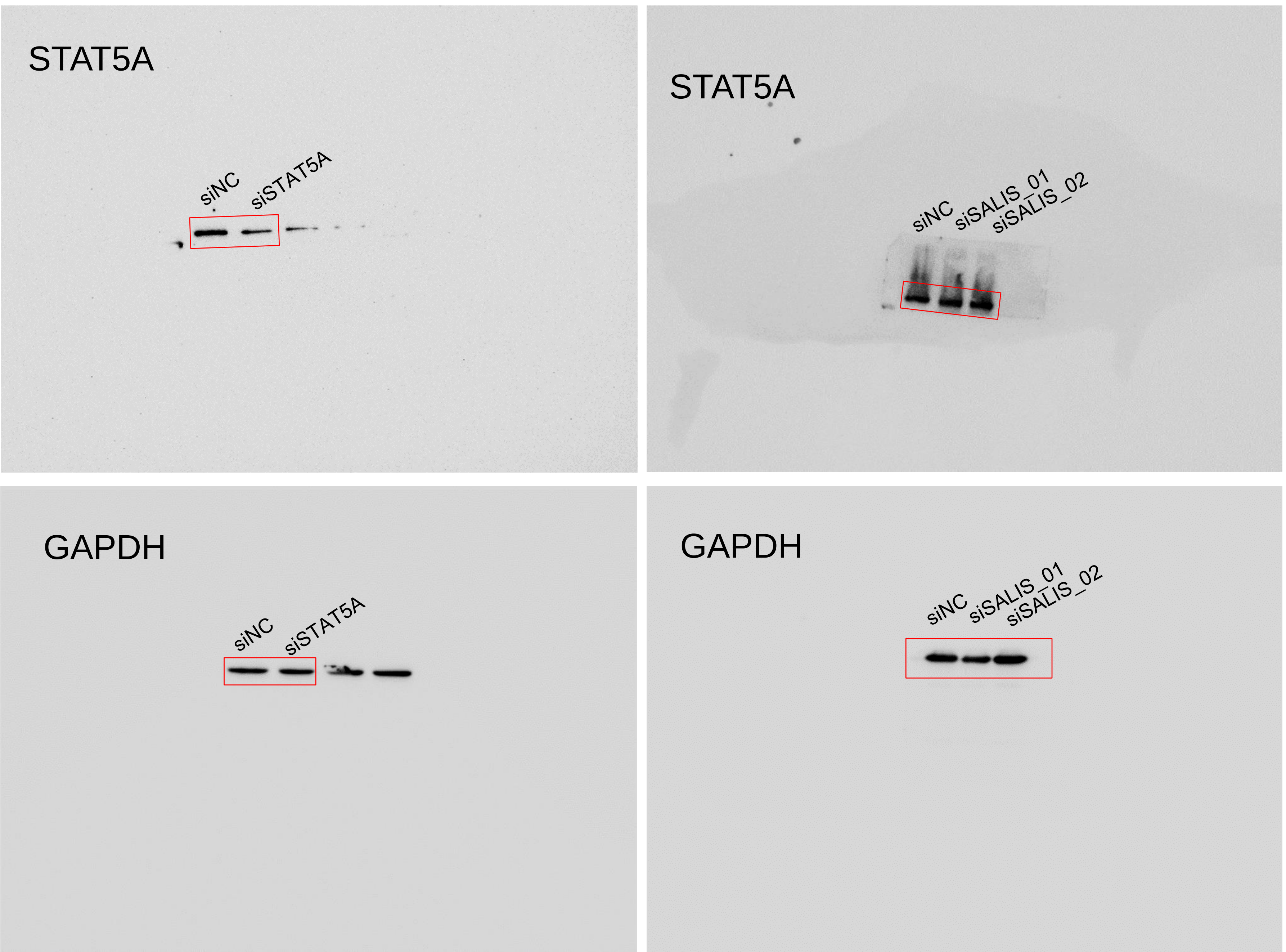

Related to Supplementary Figure S5d

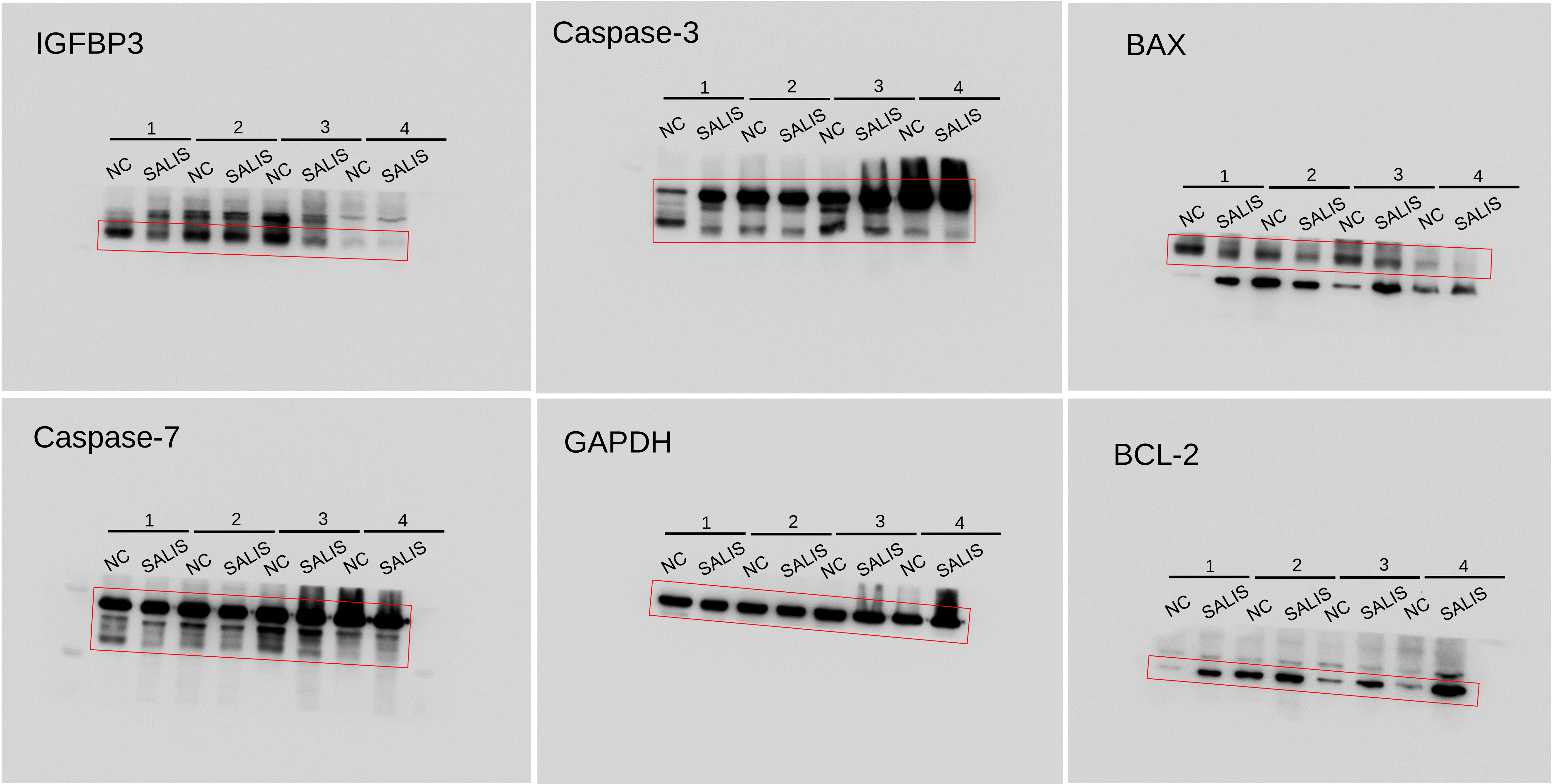

Supplement: Supplementary file 6 — Original Data File [file 41419_2022_5094_MOESM6_ESM.pdf]
